# DNA hypermethylation: A novel mechanism of CREG gene suppression and atherosclerogenic endothelial dysfunction

**DOI:** 10.1016/j.redox.2020.101444

**Published:** 2020-01-31

**Authors:** Yanxia Liu, Xiaoxiang Tian, Shan Liu, Dan Liu, Yang Li, Meili Liu, Xiaolin Zhang, Chenghui Yan, Yaling Han

**Affiliations:** Department of Cardiology and Cardiovascular Research Institute, General Hospital of Northern Theater Command, Shenyang, China

**Keywords:** Epigenetics, CREG, Atherosclerosis, Endothelium, Nitric oxide

## Abstract

**Objective:**

Cellular repressor of E1A-stimulated genes (CREG), a vasculoprotective molecule, is significantly downregulated in atherosclerotic vessels through unclear mechanisms. While epigenetic regulation is involved in atherosclerosis development, it is not known if the CREG gene is epigenetically regulated. The aim of this study was to assess the potential role of CREG methylation in contributing to atherosclerosis.

**Approach and results:**

Overexpression of DNA methyltransferase (DNMT)3B significantly inhibited CREG expression in human umbilical vein endothelial cells (HUVECs) and human coronary aortic endothelial cells (HCAECs).Conversely, inhibition of DNA methylation with 5-aza-2′-deoxycytidine (5-aza-dC) dose-dependently increased CREG expression. A CREG promoter analysis identified +168 to +255 bp as a key regulatory region and the CG site at +201/+202 bp as a key methylation site. The transcription factor GR-α could bind to the +201/+202 bp CG site promoting CREG transcription, a process significantly inhibited by DNMT3B overexpression. Treatment of cells with oxidized low-density lipoprotein (ox-LDL), a critical atherosclerogenic factor, significantly increased DNMT3B expression, increasing CREG promotor methylation, blocking GR-α binding, and inhibiting CREG expression. Consistently, CG sites in the CREG promoter fragment were hyper-methylated in human atherosclerotic arteries, and CREG expression was significantly reduced. A negative correlation between DNMT3B and CREG expression levels was observed in human atherosclerotic arteries. Finally, Ox-LDL-induced endothelium dysfunction was significantly attenuated by both 5-aza-dC and an anti-oxidative molecular N-acetylcysteine (NAC) administration through rescue the expression of CREG and activation of the p-eNOS/NO pathway.

**Conclusions:**

Our study provides the first direct evidence that DNMT3B-mediated CREG gene hypermethylation is a novel mechanism that contributes to endothelial dysfunction and atherosclerosis development. Blocking CREG methylation may represent a novel therapeutic approach to treat ox-LDL-induced atherosclerosis.

## Highlights

• Overexpression of DNMT3B but not DNMT1 or DNMT3A in HUVECs inhibits CREG expression.
• The CREG promoter contains a key methylation CG site at +201/+202 bp.
• GR-α can bind to the +201/+202 bp site to promote CREG transcription.
• Blocking CREG methylation may represent a novel therapeutic approach to treat ox-LDL-induced atherosclerosis.

## Non-standard abbreviations and acronyms

CREG: Cellular repressor of E1A-stimulated genes
HUVECs: human umbilical vein endothelial cells
ox-LDL: oxidized low-density lipoprotein
DNMT: DNA methyl transferase
HCAECs: Human coronary artery endothelial cells
NO: nitric oxide
ROS: reactive oxygen species
p-eNOS: phospho-endothelial NO synthase
NOx: nitrate/nitrite
HE: Hematoxylin-Eosin
TSS: transcription start site
ATG: translation start site
NAC: N-acetylcysteine
MOI: multiplicity of infection
5-aza-dC: 5-aza-2′-deoxycytidine
TF: transcription factor
RLU: relative light unit
ChIP: chromatin immunoprecipitation
TBARS: thiobarbituric acid-reactive substance
Ach: acetylcholine
DCF-DA: 2′, 7′– dichlorofluorescin diacetate
DAPI: 4′, 6-diamidino-2-phenylindole
t-eNOS: total endothelial NO synthase
mCREG: mouse CREG gene

## 1 Introduction

Cellular repressor of E1A-stimulated genes (CREG) is a ubiquitously expressed glycoprotein that is highly expressed in blood vessels under physiological conditions, and at very low levels in atherosclerotic vessels [1,2]. Our previous studies have demonstrated that CREG is abundant in both the arterial endothelium and in primary human umbilical vein endothelial cells (HUVECs) [3]. Overexpression of CREG not only accelerates endothelial cell proliferation and migration, but can also inhibit endothelial apoptosis and promote vasculogenesis [[3], [4], [5]]. Furthermore, overexpression of CREG can attenuate atherosclerotic plaque formation through the suppression of macrophage inflammation in an ApoE^-/-^ mouse model [1]. However, two critical questions remain unanswered that prevent a full understanding of the role CREG in vascular hemostasis. First, the question as to how atherosclerogenic factors, such as oxidized low-density lipoprotein (ox-LDL), inhibit CREG expression remains unknown. Second, detailed molecular mechanisms responsible for the CREG-mediated improvement of atherosclerosis remain unclear.

As a bridge between genes and the environment, epigenetic changes are known to be involved in the modulation of genes related to atherosclerosis. One of the major epigenetic changes is DNA methylation which is a major regulator of chromatin structure and function [6,7]. DNA methylation mainly occurs at palindromic CG dinucleotides in mammals. DNA methylation is generally catalyzed by DNA methyltransferases (DNMTs) which catalyze the addition of methyl groups to the C5 position of cytosine residues, and are typically associated with transcriptional repression. Three DNMT enzymes contribute to the generation and maintenance of DNA methylation patterns: DNMT1 exhibits a strong preference for hemi-methylated CpG sites, whereas DNMT3A and DNMT3B methylate unmethylated and hemi-methylated DNA sites equally and can also catalyzed non-CpG methylation. DNA methylation in promoter regions is associated with changes in gene expression and silencing. It is thought that aberrant DNA methylation may underlie pathogenic conditions such as atherosclerosis [8,9]. A previous study performed in culture cells suggested that expression at the CREG locus might be epigenetically modified by DNA methylation [10]. However, critical questions remain unanswered. First, the question as to whether atherosclerotic factors, such as ox-LDL, result in epigenetic CREG suppression by DNA methylation has not been previously investigated. Second, the specific DNA regions that is (are) methylated must be identified. Third, the clinical relevance of epigenetic CREG suppression by DNA methylation has never been previously investigated.

Therefore, the aims of the present study were 1) to determine whether CREG gene expression is regulated by DNA methylation; 2) to identify the specific regions whose methylation is responsible for CREG gene suppression, and 3) to clarify whether and how DNA methylation-induced CREG gene suppression may impair endothelial function.

## 2 Materials and methods

### 2.1 Cell culture

HUVECs were isolated from umbilical cord veins and cultured as previously described [11]. HUVEC isolation was performed in accordance with the declaration of Helsinki and approved by the ethics review board at the General Hospital of Northern Theater Command, Shenyang. Cells at passage 2–5 were used in this study.

The primary human coronary artery endothelial cells (HCAECs) used for pyrosequencing were isolated from human coronary arteries (as follows) and cultured with an agarose microsphere antibody screening method as previously described [12].An inverted microscope was used to observe the cell morphology, and immune-fluorescence staining for the CD31 and vWF related antigen was performed to identify the cells.

The HCAECs for the other tests were purchased from ScienCell Research Laboratories (San Diego, California).

### 2.2 Patients and blood vessels

Lower extremity arterial blood vessels were obtained from amputees, including 8 individuals who underwent lower extremity amputations, 3 individuals with coronary atherosclerosis, and individuals without atherosclerosis who had suffered a traffic accident (n = 8) or other accident and underwent an autopsy (n = 3). The use of human vessels was approved by the Ethics Committee of the General Hospital of Northern Theater Command of Shenyang Military Region and informed consent was obtained from all patients. Collected human diseased and control arteries were treated appropriately for analysis of nitric oxide (NO) concentration, reactive oxygen species (ROS) production, CREG, and phospho-endothelial NO synthase (p-eNOS), expression using a Nitrite/Nitrate (NOX) Assay Kit, a ROS Assay Kit, arterial diastolic dysfunction analysis, and hematoxylin-eosin (HE) and immunostaining.

### 2.3 Promoter CpG Island Search

The properties of CpG islands in the proximal region of *CREG* (-1000/+1000 bp) were examined using a CpG Island Search engine (http://www.urogene.org/cgi-bin/methprimer/methprimer.cgi). A CpG island was defined as a DNA region >200 bp with a CG content of≥50% and CpG ratio of≥0.6 [13].

### 2.4 Adenoviral infection

Adenoviral vectors containing the *DNMT3A* (Ad-DNMT3A) genes were constructed by Genomeditech, Shanghai. Adenoviral vectors containing *DNMT1* (Ad-DNMT1) and *DNMT3B* (Ad-DNMT3B) were constructed by OBiO Technology, Shanghai. HUVECs or HCAECs were infected with adenoviral vectors at an MOI of 100 PFU/cell for 48 h and adenovirus carrying empty vector (Ad-GFP, Genomeditech) was used as a negative control. Adenovirus-mediated gene transfer was carried out as previously described [14]. The maximal expression efficiency of transfected proteins was assessed by western blotting.

### 2.5 Real-time PCR and western blotting

Real-time PCR was performed using an ABI 7300 Real-Time PCR system (Applied Biosystems, Foster City, CA, USA), as previously described [15].The primers used are listed in Supplemental Table 1.

For western blot analysis, cell homogenates were lysed in RIPA buffer (Thermo Scientific, Waltham, MA, USA) containing protease and phosphatase inhibitors. Cleared supernatants were collected and protein concentrations were determined using a BCA Protein Assay Kit (Thermo Scientific, Waltham, MA, USA). The levels of CREG, p-eNOS (Ser1177), eNOS, DNMT1, DNMT3A, DNMT3B, GR-α, and GAPDH were determined by western blotting specific antibodies (Cell Signaling Technology, Danvers, MA or Sigma Aldrich, St.Louis, MO, USA). Western blotting was performed as previously described [14].

### 2.6 *CREG* CpG reporter gene constructs and promoter activity assay

Various fragments from the 5′-flanking region of *CREG*, containing 675 bp (−508/+167), 763 bp (−508/+255), 940 bp (−508/+432), 1096 bp (−508/+588), and 1202 bp (−508/+694), with XhoI/HindIII restriction sites, were either synthesized or amplified by PCR (Takara, China). The core promoter element of *CREG* (−508/+78 bp) was synthesized as previously described [11]. The transcription start site (TSS) was designated as “+1” throughout the text, with the translation start site (ATG) at position +78 bp. All fragments were cloned into the pGL4.12-Basic promoter dual luciferase reporter plasmid (Promega, Madison, WI, USA), and sequenced. The promoter activity of the *CREG* constructs was evaluated in cultured HUVECs and 293T cells by transient transfection and luciferase assay, as previously described [11]. A Renilla luciferase expression plasmid, pGL4.73 (0.02 μg), was co-transfected to correct for variability in transfection efficiency. The promoter activities of reporter constructs were normalized to that of pGL4.73 and are expressed as fold-increase relative to that in cells transfected with pGL4.12_-508/+78.

To determine the functional importance of consensus elements in the key CpG island of the *CREG* promoter, CG sequences were mutated to AT using a site-directed mutagenesis kit from Invitrogen (Frederick, MD, USA). Mutant fragments were then cloned into reporter vectors and promoter activity determined as described above. Each construct was transfected six times and each transfection was performed in triplicate.

### 2.7 Bisulfate genomic DNA sequencing

HUVECs were plated on 100-mm plates (2–5 × 10^5^ cells/cm^2^) and cultured to 80%–90% confluence. The culture medium was changed to untreated control medium or medium containing 50 multiplicity of infection (MOI) Ad-DNMT3B for 48 h, 5 μM 5-aza-2′-deo- xycytidine (5-aza-dC) for 72 h, 40 μg/mL ox-LDL or 1mM N-acetylcysteine (NAC) for 24 h respectively. Genomic DNA was extracted and modified by bisulfite treatment, which converts all unmethylated cytosines to uracil, using the EpiTect bisulfite kit (Qiagen, Valencia, CA, USA), according to the manufacturer's instructions. PCR products from bisulfite-treated genomic DNA samples were analyzed using pyrosequencing technology to quantify site-specific methylation. Sequencing samples were prepared using a Vacuum Prep workstation (Biotage AB, Uppsala, Sweden). Pyrosequencing was performed using the PyroMark Gold Q96 Reagent and the PyroMark ID system (Qiagen, Germany). The sequencing primers used are listed in Supplemental Table 2. During assay design, Pyro Q-CpG™ software v. 1.0.9 was used to determine the optimal order of nucleotide addition. This software also automatically analyzed the methylation results. The pyrosequencing analysis was performed at Sangon Biotech Co., Ltd. (Shanghai, China).

### 2.8 Promoter-binding transcription-factor (TF) profiling array assay

To screen for TFs that bind to the *CREG* key CpG island (+200/+255 bp), the activities of 48 TFs in primary HUVECs were assayed using a Promoter-Binding TF Profiling Array (Signosis, Santa Clara, CA, USA), as previously described [16]. Luminescence is reported as relative light units (RLUs), measured using a microplate luminometer (Wallac 1450, Wallac, MA, USA). Assays were performed as recommended in the manufacturer's instructions.

### 2.9 Chromatin immunoprecipitation (ChIP) assay

ChIP assays were performed using a ChIP assay kit, as per the manufacturer's instructions. The PCR primers used to amplify the predicted GR-α binding sequence in the *CREG* key CpG island (+200/+255 bp) were: 5′- GACTCTTCCTGGAGACACCG -3′ (forward) and 5′- GTGGTAGCGGCGGCAG -3′ (reverse).

### 2.10 Preparation of ox-LDL

Human LDL was purchased from Sigma and dialyzed against PBS (50 mM phosphate buffer, pH 7.4, 0.15 M NaCl, 0.01% NaN_3_ and 6.67μM CuSO_4_) at 37 °C for 24 h. After the incubation was terminated by EDTA (0.5 mg/mL), the preparations were dialyzed and preserved in nitrogen-filled tubes. The extent of LDL oxidation was determined using the thiobarbituric acid-reactive substance (TBARS) test [17].

### 2.11 Arterial ring relaxation

Human arterial rings were prepared and pre-contracted using 30 nM U46619 and endothelium-dependent relaxation was induced by the addition of acetylcholine (Ach) at 10^−9^ to 10^−5^ M, as previously described [18]. Vaso-relaxation was expressed as the percentage dilation of U46619-induced pre-constriction. Arteries were contracted using U46619 (30 nM) to produce consistent submaximal (~90%) responses. After equilibration, the responsiveness and stability of individual rings were checked by successive administration of sub-maximal effective concentrations of U46619. The integrity of the vascular endothelium was assessed pharmacologically using Ach to induce relaxation of U46619-precontracted rings. Arteries that did not elicit a reproducible and stable contraction with U46619 (relaxation < 10% with 10^-7^ M Ach) were eliminated from the study. Aortic rings were considered denuded of functional endothelium when there was no relaxation response to Ach.

### 2.12 Measurement of nitric oxide concentration

HUVECs or HCAECs culture medium was collected and assayed for NO concentration using a Nitrite/Nitrate Assay Kit (Sigma Aldrich,St.Louis, MO, USA), according to the manufacturer's instructions. Tissue NOx levels were measured in arteries taken from amputees with low extremity atherosclerosis or individuals without atherosclerosis as described above. NOx levels are expressed in nmol/g protein as previously described [19].

### 2.13 Assessment of ROS production

Intracellular ROS production was detected by staining with 2′, 7′– dichlorofluorescin diacetate (DCF-DA), a ROS-sensitive fluorescent dye (Beyotime Institute of Biotechnology, Nanjing, China) [20]. Briefly, HUVECs or HCAECs pretreated with 40 μg/mL ox-LDL for 24 h were washed with phosphate buffered saline and loaded with freshly prepared DCF-DA (10 μM) for 15 min at 37 °C. 4′, 6-diamidino-2-phenylindole (DAPI) was used to stain nuclei. DCF-DA signals were recorded by fluorescence microscopy. Lower extremity arterial cryosections (8 mm) were stained with DCF-DA probes and DAPI as previously described [20].

### 2.14 Statistical analysis

Data are expressed as the mean ± SE. Statistical analyses were performed using SPSS version 20.0 (SPSS, Inc., Chicago, Illinois). A Student's *t*-test was used for comparisons between two groups, and a one-way ANOVA with Tukey's post hoc analysis was used to compare more than two groups. P values < 0.05 were considered statistically significant.

## 3 Results

### 3.1 DNMT3B regulates the expression of CREG in HUVECs

**Fig. 1:**
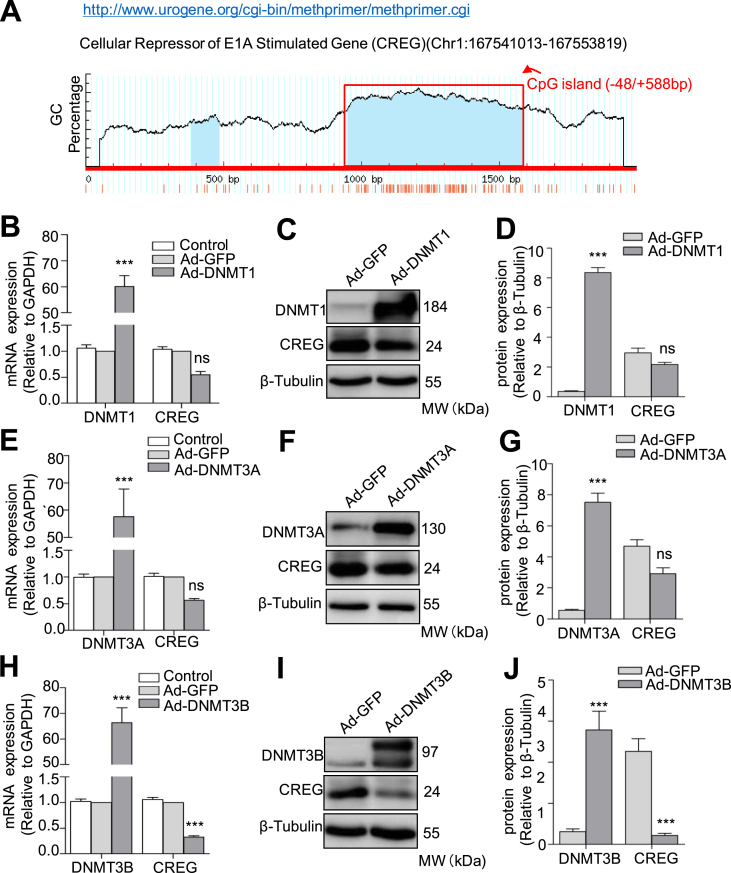
**DNMT3B directly regulates the expression of CREG in HUVECs.** (A) One CpG island (-48/+588bp) was identified in the core promoter region of the CREG gene by computational analysis. (B to J) Quantitative real-time PCR (B, E, H), representative western blotting (C, F, I), and quantification analysis (D, G, J) of CREG, DNMT1, DNMT3A and DNMT3B in HUVECs after infection with Ad-DNMT1, Ad-DNMT3A, and Ad-DNMT3B for 48 h respectively. Specific proteins were quantified in western blots using Image-Pro plus software. Data are presented as the mean ± SE. ns, no significant difference, n = 6 per group, **P < 0.01, and ***P < 0.001 vs. Ad-GFP; two-sided Student's *t*-test.

Endothelial dysfunction contributes to the initiation and development of atherosclerosis and may be rescued by CREG expression. Therefore, we attempted to determine why CREG mRNA expression is down-regulated in atherosclerotic endothelium. To address this question, we analyzed the DNA sequence of the 5′-flanking region of the human *CREG* gene (-1,000 to +1000 bp) accessed via the NCBI database. Interestingly, two CpG islands were detected in this region. A primary CpG island was identified from nucleotides -48 to +588 bp (CG content, 74.1%; CpG ratio, 0.95) as shown in Fig. 1A. To investigate whether DNA methylation is related to the expression of CREG, we overexpressed DNMT1, DNMT3A, or DNMT3B in HUVECs by adenovirus infection to mimic hyper-methylation, and then evaluated changes in CREG expression. Real-time PCR (Fig. 1B, 1E, and 1H) and western blotting (Fig. 1C, 1F, and 1I) showed that increased levels of DNMT3B, but not DNMT1 or DNMT3A, significantly inhibited CREG expression at both the mRNA and protein levels in HUVECs. To confirm this result, we further transiently transfected *CREG* promoter reporter vector (pGL4.12_-508/+588) into HUVECs, and then infected them at a MOI of 50 with Ad-DNMT1, Ad-DNMT3A, or Ad-DNMT3B for 48 h respectively. A luciferase activity analysis also revealed that overexpression of DNMT3B dramatically decreased the expression of the reporter gene compared to that in HUVECs overexpressing DNMT1 or DNMT3A (Fig. 1K). Conversely, when we used 5-aza-dC (0, 1, 5, and 10 μM), a DNA methylation inhibitor, to inhibit *DNMT3B* expression, CREG levels gradually increased at both the mRNA and protein levels in HUVECs in a dose-dependent manner (Supplement Fig. 1A to 1C). These data suggest that DNMT3B is an important regulator of CREG expression in HUVECs.

### 3.2 CG (+201/+202 bp) is a key DNA methylation site in the CREG promoter

**Fig. 2:**
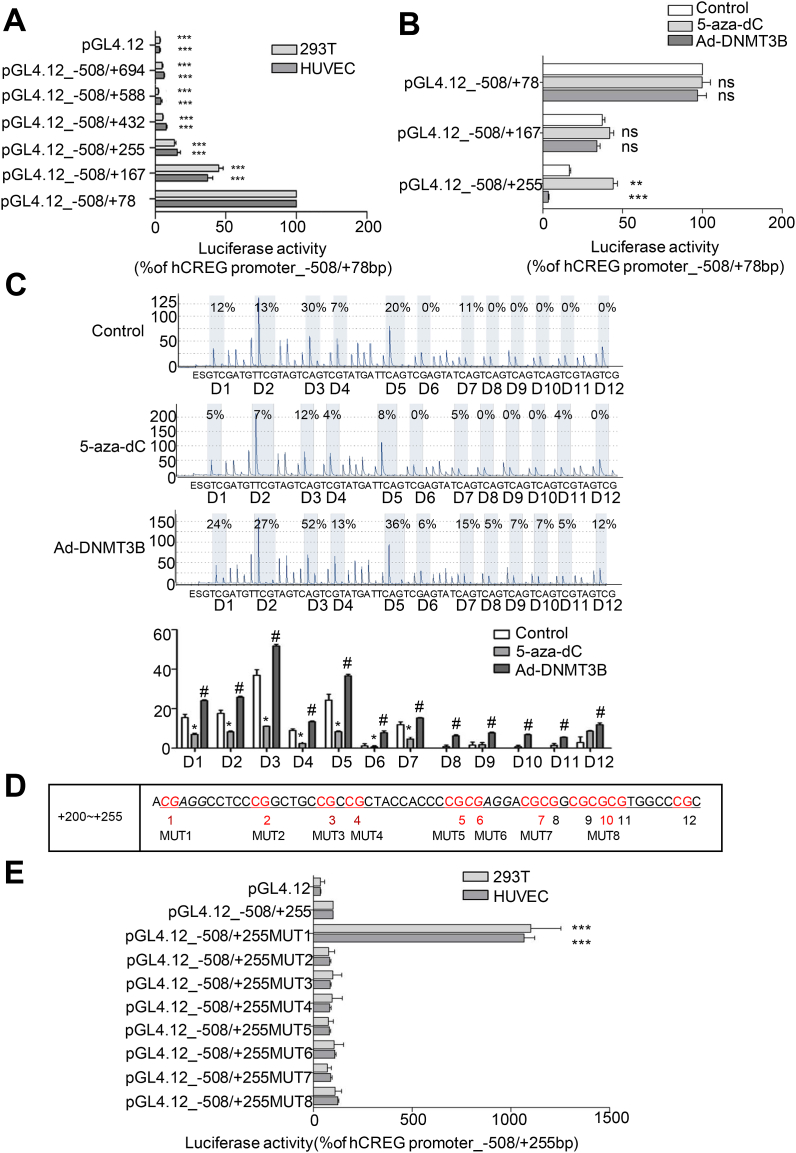
**Determination of the key DNA methylation regulatory sites in the CREG promoter region in vitro.** (A) The CREG core promoter is located at -508/+78bp. A series of CpG island deletions were created, based on this core promoter, and then subcloned into the pGL4.12-basic luciferase reporter vector. HUVECs and 293T cells were transiently transfected with reporter vectors and harvested after 48 h. For each construct, a pGL4.73 plasmid was co-transfected, to correct for differences in transfection efficiency. Luciferase activity was normalized to that using the plasmid containing CREG -508/+78bp (100% activity). Data are shown as the mean ± SE from six independent experiments performed in triplicate,n = 9 per group, ***P < 0.001 compared with the CREG core promoter (100%). (B) HUVECs were transiently transfected with the key reporter vectors using Lipofectamine 3000 and treated with 5 μM 5-aza-dC for 72 h or Ad-DNMT 3B (MOI of 50) for 48 h and compared with untreated HUVECs (negative control). Luciferase activity was normalized to that generated by the CREG-508/+78bp plasmid (100% activity). Data are the mean ± SE from three independent experiments performed in triplicate,n = 9 per group, **P < 0.01, ***P < 0.001 compared with the control group. (C) Pyrosequencing of the twelve CG sites located between +200 and +255 bp in CREG from primary HUVECs treated with 5 μM 5-aza-dC for 72 h or Ad-DNMT3B (MOI of 50) for 48 h, compared with untreated HUVECs (negative control); n = 7. Data are the mean ± SE. *, #, P < 0.05. (D) The online gene promoter region transcription factor prediction software, PROMO, was used to predict the transcription factors that may bind to CG sites in the CpG island sequence between +200 and +255 bp. (E) Plasmids containing the core promoter and the key CpG island in CREG (-508/+255 bp) with eight deletion mutations at consensus CREG biding elements were inserted upstream of the luciferase gene in the pGL4.12 vector. Luciferase activity was normalized to that of the CREG promoter (-508/+255 bp) (100%). Data are the mean ± SE from three independent experiments performed in triplicate,n = 9 per group, ***P < 0.001 compared with the core promoter and the key CpG island of CREG (-508/+255 bp) (100%).

As shown in Fig. 2A, using a series of luciferase CREG promoter reporter vectors (pGL4.12) to transfect HUVECs, a key *CREG* promoter regulatory region was localized within the fragment from +79 to +255 bp, which down-regulated transcription by approximately 85%. Furthermore, this key regulatory region used to design three additional reporter vectors (pGL4.12_-508/+78, pGL4.12_-508/+167 and pGL4.12_-508/+255). The highest level of repression (79.0%) of luciferase activity occurred in HUVECs transfected with pGL4.12_ -508/+255 following infection with by Ad-DNMT3B (Fig. 2B). Conversely, 5-aza-dC increased the luciferase activity of pGL4.12_-508/+255 by 164.1% in HUVECs compared to that of untreated cells (Fig. 2B). In contrast, the luciferase activity generated by the CREG promoter reporter plasmids pGL4.12_-508/+167 and pGL4.12_-508/+78 was not significantly influenced by either Ad-DNMT3B or 5-aza-dC treatment (Fig. 2B). These data indicate that the fragment of the *CREG* promoter from +168 to +255 bp is a key regulatory region that is regulated by DNA methylation.

To test the above result, we next generated three PCR fragments (+74/+128 bp, +129/+199 bp and +200/+255 bp) derived from primary HUVECs pretreated with Ad-DNMT3B (MOI of 50) or 5-aza-dC (5 μM) and conducted pyrosequencing. Similar to the data shown in Fig. 2B, there was no significant difference in the methylation levels of the first (+74/+128 bp) or the second (+129/+199 bp) PCR fragments between the treated and control groups (Supplemental Figs. 2A and 2B). However, the amplified fragment (+200/+255 bp) was highly methylated in the Ad-DNMT3B-treated HUVECs, while a lower level of methylation was detected in 5-aza-dC-treated HUVECs compared with untreated HUVECs (n = 7) (Fig. 2C and Supplemental Fig. 2C). Moreover, CG sites (D1–D7) in the fragment were hypomethylated following treatment with 5-aza-dC, whereas the CG sites D1–D12 showed hypermethylation after treatment with Ad-DNMT3B compared with the control group.

To determine which methylation site is more important in *CREG* transcription, we constructed a further eight mutated plasmids in which CG was replaced by AT in the consensus *CREG* binding element (+200/+255 bp) as shown in Fig. 2D. Compared to the wild-type vector pGL4.12_- 508/+255, the transcriptional activity of the first CG mutated plasmid (pGL4.12_-508/+255 MUT1) was dramatically increased by 11.0-fold (Fig. 2E). There were no significant differences in the activities of the other mutated constructs compared with the control fragment, suggesting that the CG site at position +201/+202 bp is a key methylation regulatory site in the *CREG* promoter.

### 3.3 GR-α binds to CG sites (+201/+202) to promote the transcription of CREG

**Fig. 3:**
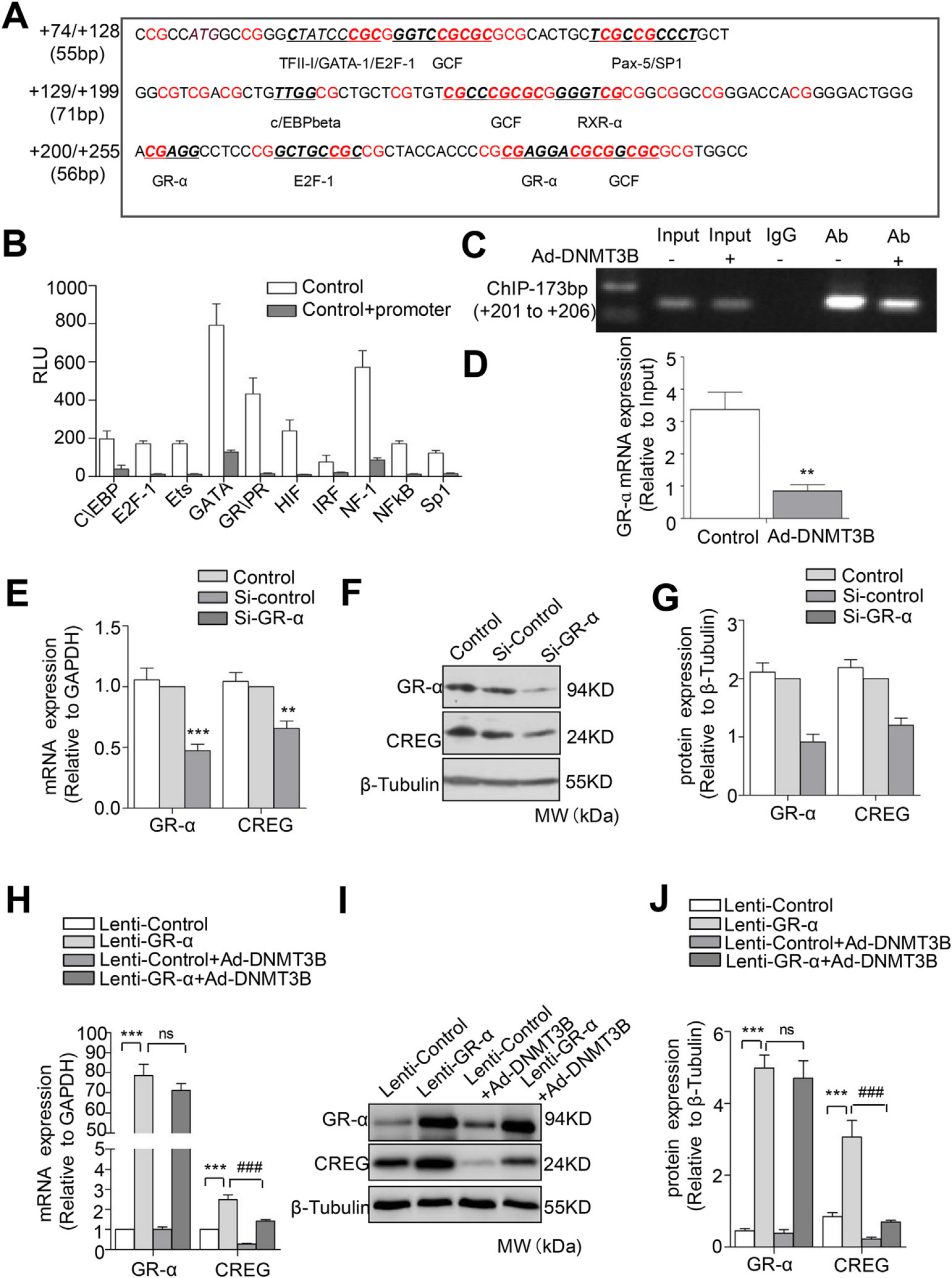
**GR-α directly binds to CG site (+201/+202) to promote the transcription of CREG.** (A) There are 35 CG sites (red) between CREG +79 and +255 bp. The online gene promoter region transcription factor prediction software, PROMO, was used to predict transcription factors that may bind to sites in the CpG island sequence. (B) A promoter-binding transcription-factor (TF) profiling array assay of the CREG core promoter and the key CpG islands of CREG (-508/+255bp),n = 3. (C, D) ChIP analysis of CREG promoter in HUVECs stimulated Ad-DNMT3B for 48 h. Cross-linked chromatin was immunoprecipitated with an antibody to GR-α, in the absence of antibody (input), or an isotype-matched control (IgG). Isolated DNA was purified and analyzed by PCR. Data are representative of 3 independent experiments. (E) Quantitative real-time PCR, (F) representative western blotting, and (G) quantification in primary HUVECs with GR-α knocked down using siRNA (Si-GR-α). (H) Quantitative real-time PCR, (I) western blotting, and (J) quantification in primary HUVECs overexpressing GR-α (Lenti-GR-α) with or without Ad-DNMT3B infection. Quantification of western blots was conducted using Image-Pro plus software. Data are presented as the mean ± SE, n = 6 per group, **P < 0.01 and ***, ^###^*P* < 0.001 vs. Lenti-control, or Si-control groups. (For interpretation of the references to colour in this figure legend, the reader is referred to the Web version of this article.)

To elucidate the mechanism by which DNA methylation controls the transcription of *CREG*, we attempted to identify potential TFs that could bind to the *CREG* promoter (+200/+255 bp). An analysis with the MethPrimer software revealed several potential TF binding sites, including GR-α, E2F-1, and GCF in this region. In particular, GR-α was found to bind to the +201/+206 site in the C*REG* promoter (Fig. 3A). We also used a competitive promoter-binding TF profiling array to assess the binding of 48 TFs to the *CREG* promoter in HUVECs. TFs found to bind to the *CREG* promoter were C\EBP, E2F-1, ETS, GATA, GR/PR, HIF, IRF, NF-1, NF-KB, and Sp1 (Fig. 3B). Subsequently, we conducted a ChIP assay, which confirmed that GR-α can bind directly to *CREG* at the consensus GR-α binding sequence at +201/+206 bp (Fig. 3C). A quantitative assay demonstrated that GR-α binding to the *CREG* promoter fragment was reduced by 73.1% in Ad-DNMT3B treated cells compared with control cells (Fig. 3D).

To reveal whether GR-α mediated DNA methylation-regulated *CREG* transcription, gain/loss-of-function assays were conducted following infection with lentiviruses expressing a siRNA targeting GR-α or a GR-α vector in HUVECs. As shown in Fig. 3E–G, HUVECs infected with a lentivirus expressing an siRNA targeting GR-α (Si-GR-α) exhibited a marked decrease in CREG expression at both the mRNA (Fig. 3E; n = 6) and protein (Fig. 3F and G; n = 6) levels. In contrast, the expression of CREG dramatically increased at both the mRNA (Fig. 3H; n = 6) and protein levels (Fig. 3I and J; n = 6) in HUVECs infected with lentivirus expressing GR-α (Lenti-GR-α) compared to those infected with a control lentivirus (Lenti-control). Once infected with Ad-DNMT3B, the increased expression of CREG was inhibited both in the Lenti-control and Lenti-GR-α cells (Fig. 3H–J). Furthermore, immunofluorescent staining demonstrated that CREG expression was decreased in HUVECs when GR-α expression was knocked down (Supplemental Fig. 3A) and was up-regulated following overexpression of GR-α (Supplemental Fig. 3B). These data indicate that GR-α mediates DNA methylation-regulated *CREG* transcription by directly binding to the CG (+201/+202) site in the *CREG* promoter region.

### 3.4 5-Aza-dC administration rescued the ox-LDL-induced repression of CREG to improve HUVEC dysfunction

**Fig. 4:**
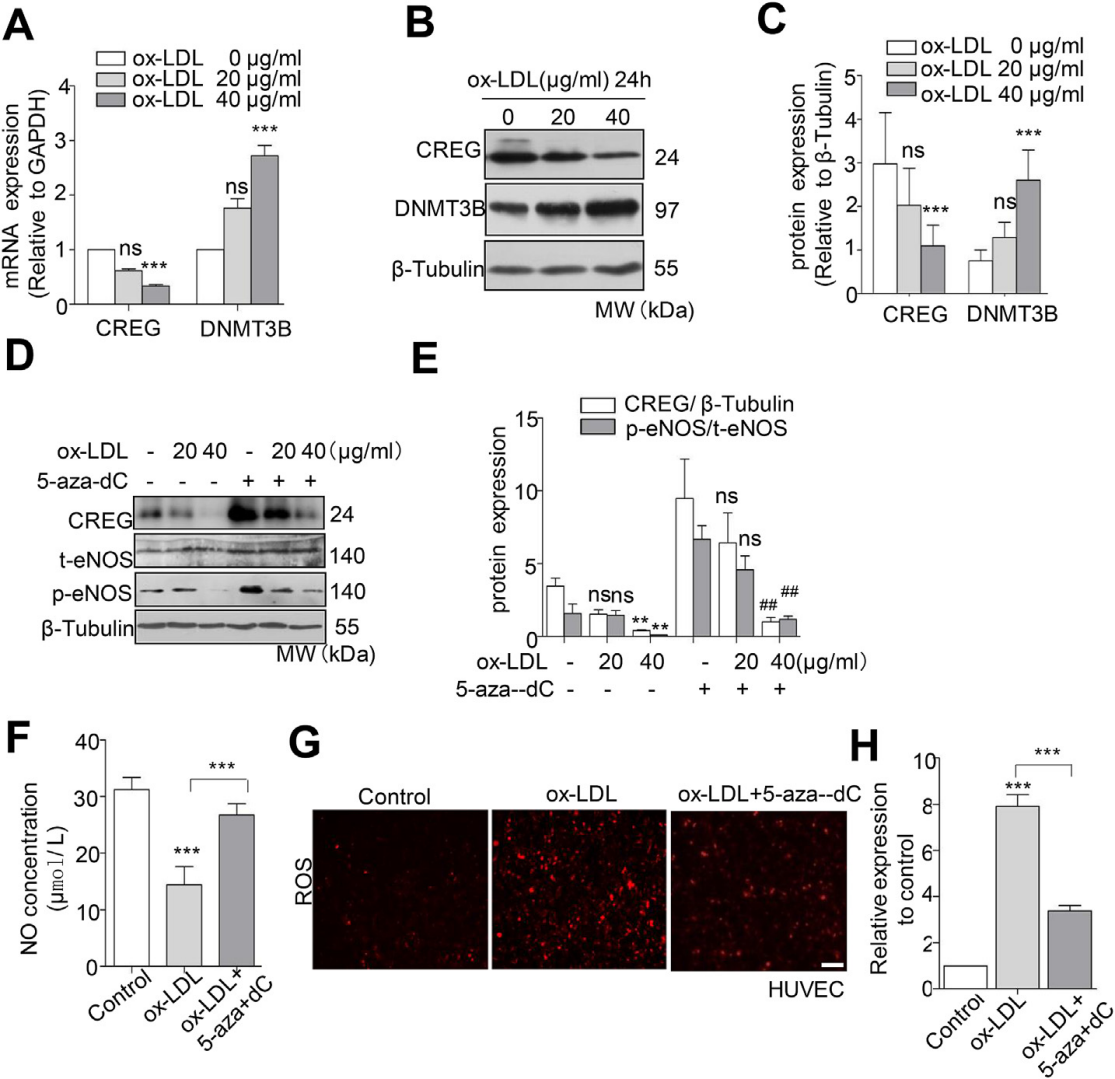
**5-aza-dC up-regulates CREG to inhibit the ox-LDL-induced HUVEC impairment.** (A) Quantitative real-time PCR, (B) representative western blotting, and (C) quantification of CREG and DNMT3B in primary HUVECs treated with Ad-DNMT3B. Quantification of western blots was conducted using Image-Pro plus software. Data are presented as the mean ± SE, n = 6 per group, *P < 0.05, ***P < 0.001 vs ox-LDL (0 μg/mL) (D) Representative western blot and (E) quantification of CREG and eNOS in primary HUVECs exposed to 20 or 40 μg/mL ox-LDL in the presence or absence of 5 μM 5-aza-dC for 72 h, n = 6 per group, **P < 0.01 vs. ox-LDL(-) + 5-aza-dC(-), ^##^P < 0.01 vs. ox-LDL (40 μg/mL) + 5-aza-dC(-), (F) NO concentration and (G) ROS production in HUVECs induced by ox-LDL (40 μg/mL) for 24 h and in the presence of 5 μM 5-aza-dC for 72 h (n = 6, ***P < 0.001). (H) Semi-quantitative analysis of immunostaining using Image-Pro plus software.Scale bars, 100 μm. Data are presented as the mean ± SE, n = 6 per group, ***P < 0.001 vs control group.

Having demonstrated that GR-α-mediated DNA methylation regulates CREG transcription, we further determine whether atherosclerotic factors may result in epigenetic CREG suppression by DNA methylation, and more importantly, whether this inhibition can be rescued by 5-aza-dC administration. Ox-LDL is a crucial initiation factor in atherosclerosis, which accumulates in the vascular endothelium and contributes to endothelial dysfunction by inhibiting NO production and inducing DNA methylation [[21], [22], [23]]. Thus, we used ox-LDL treatment to mimic hypermethylation and then evaluated the expression of CREG in HUVECs. As expected, treatment with ox-LDL dramatically decreased CREG expression and increased levels of DNMT3B in a dose-dependent manner at both the mRNA (Fig. 4A) and protein levels (Fig. 4B and 4C). Meanwhile, 5-aza-dC treatment blocked the ox-LDL-induced reduction of both CREG and p-eNOS1177 expression in a dose-dependent manner. However, there was no change in total endothelial NO synthase (t-eNOS) expression in HUVECs following either ox-LDL or 5-aza-dC treatment (Fig. 4D and 4E). Furthermore, 5-aza-dC treatment rescued the ox-LDL-induced reduction of NO (Fig. 4F) and increased ROS production (Fig. 4G and 4H).

### 3.5 NAC upregulates CREG to inhibit ox-LDL-induced HUVEC impairment

Because ox-LDL has been reported to enhance the formation of intracellular ROS, we next investigated whether inhibiting the redox state can rescue CREG expression and endothelial dysfunction. Interestingly, the increases in the ox-LDL-induced DNMT3B expression and CREG promotor methylation were attenuated by cotreatment with an anti-oxidative molecule NAC (1 mM) for 24 h.

**Fig. 5:**
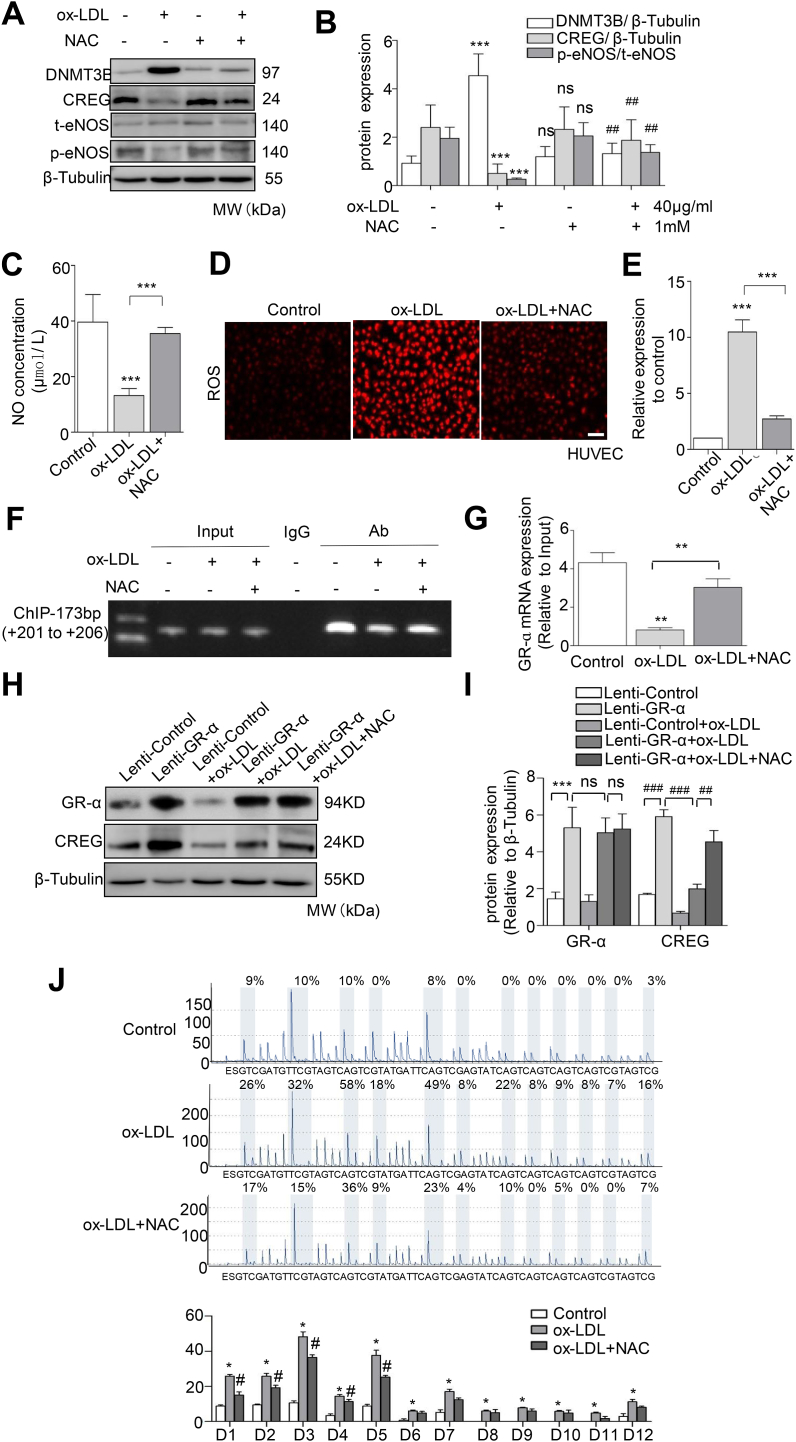
**NAC upregulates CREG to inhibit ox-LDL-induced HUVEC impairment.** (A) Representative western blotting and (B) quantification of DNMT3B, CREG and eNOS in HUVECs exposed to 40 μg/mL ox-LDL alone or in combination with 1mM NAC for 24 h, n = 3 per group, ***P < 0.001 vs. ox-LDL(-) + NAC(-), ^##^P < 0.01 vs. ox-LDL (40 μg/mL) + NAC(-). (C) NO concentration and (D) ROS production in HUVECs induced by ox-LDL (40 μg/mL) for 24 h and in the presence of 1mM NAC for 24 h (n = 3, ***P < 0.001). (E) Semi-quantitative analysis of immunostaining using Image-Pro plus software. Scale bars, 100 μm. Data are presented as the mean ± SE, n = 3 per group, ***P < 0.001 vs control group or ox-LDL. (F, G) ChIP analysis of CREG promoter in HUVECs stimulated with ox-LDL and/or NAC for 24 h. Cross-linked chromatin was immunoprecipitated with an antibody to GR-α, in the absence of antibody (input), or an isotype-matched control (IgG). Isolated DNA was purified and analyzed by PCR. Data are representative of 3 independent experiments.(H) Representative western blotting and (I) quantification of GR-α and CREG in HUVECs exposed to GR-α and/or ox-LDL or in combination with NAC for 24 h, n = 3 per group, ^##^P < 0.01, ***^,###^P < 0.001.(J) Pyrosequencing of the twelve CG sites located between +200 and +255 bp in CREG from primary HUVECs treated with 40 μg/mL ox-LDL or 1mM NAC for 24 h, n = 3. Data are the mean ± SE. *P < 0.05 vs untreated HUVECs (control), ^#^P < 0.05 vs ox-LDL.

Meanwhile, the expression of CREG and p-eNOS1177 expression were found to increase in HUVECs when they were cotreated with ox-LDL and NAC. However, there was no change in t-eNOS expression in HUVECs following treatment with ox-LDL alone or in combination with NAC (Fig. 5A and B). Furthermore, NAC treatment rescued the ox-LDL-induced reduction of NO (Fig. 5C) and ROS production (Fig. 5D and E).

ChIP assays and representative western blotting confirmed that NAC could restore GR-α binding to CREG at the consensus sequence (+201/+206 bp) (Fig. 5F–I). In addition, we found the methylation of CG sites D1–D5 in the amplified fragment (+200/+255) that is induced by ox-LDL was attenuated when the HUVECs were cotreated with NAC and ox-LDL (Fig. 5J and Supplemental Fig. 4).

### 3.6 DNMT3B also regulates the expression of CREG by binding to GR-α in HCAECs

To confirm that the DNMT3B-mediated CREG gene hypermethylation is a general mechanism, we further measured the expression of CREG in HCAECs with or without infection with an adenovirus overexpressing DNMT3B expression. Similar to the findings in the HUVECs, the overexpression of DNMT3B, but not DNMT1 or 3A dramatically inhibited the expression of CREG in HCAECs at both the mRNA and protein levels (Supplemental Figs. 5A–5I). Furthermore, infection with a lentivirus expressing Si-GR-α induced a marked decrease in CREG expression in HCAECs at both the mRNA and protein levels (Supplemental Figs. 6A–6C). In contrast, the expression of CREG remarkedly increased in HCAECs when they were infected with Lenti-GR-α relative to those infected with the Lenti-control. However, the increased expression of CREG was inhibited in both the Lenti-control and Lenti-GR-α cells after they were infected with Ad-DNMT3B (Supplemental Figs. 6D–6F).

### 3.7 CREG is negatively correlated with DNMT3B in human atherosclerotic arteries and vasorelaxation dysfunction is associated with the repression of CREG in human atherosclerotic endothelium

**Fig. 6:**
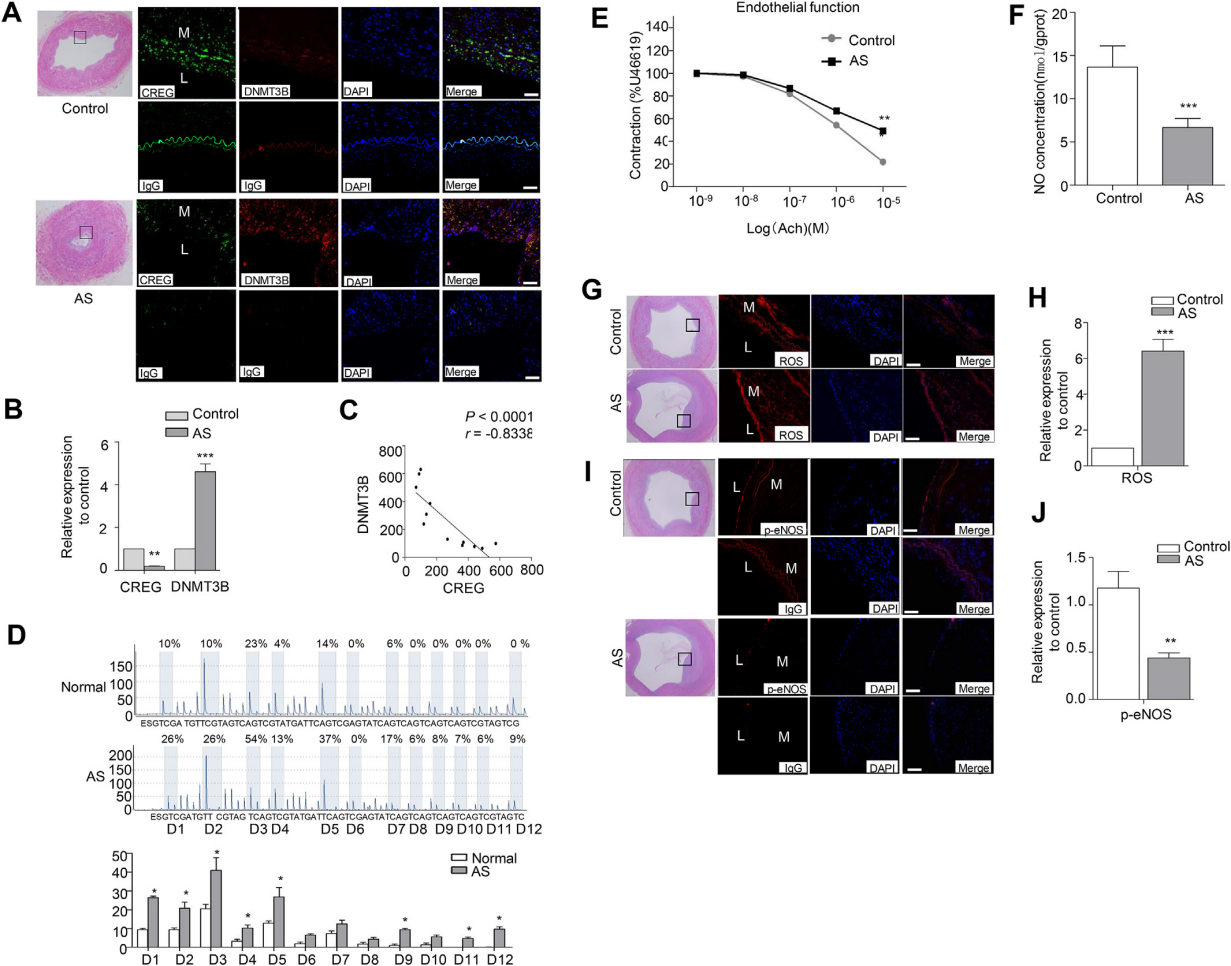
**CREG expression is negatively correlated with DNMT3B levels and vasorelaxation dysfunction is associated with repression of CREG in human atherosclerotic endothelium** (A) Representative images of HE staining (left panel) and immunostaining (right) showing CREG and DNMT3B, nonspecific IgG expression and localization in human arteries from control and patients with atherosclerosis, Green, CREG staining; Red, DNMT3B staining; Blue, cell nuclei (DAPI staining); L, lumen; M, media. Scale bar, 100 μm. (B) Semi-quantitative analysis of immunostaining using Image-Pro plus software, n = 6 per group, (C) Correlational analysis of the expression of CREG and that of DNMT3B in human arteries, n = 6 per group, *P < 0.05, ***P < 0.001 vs. normal. AS, atherosclerosis. (D) Pyrosequencing of the twelve CG sites located between +200 and +255 bp of CREG from atherosclerotic and healthy arteries; n = 11 per group. (E) Endothelial diastolic function in atherosclerotic and control vessels induced by the addition of Ach at 10^−9^ to 10^−5^ M, n = 6 per group.(F) Quantitative analysis of NO concentration in atherosclerotic and control vessels, n = 6 per group.(G) ROS production in atherosclerotic vessels and normal arteries was detected by fluorescence microscopy after DCF-DA staining. (H) Semi-quantitative analysis of immunostaining using Image-Pro plus software, n = 6 per group. (I) Representative images of immunostaining showing p-eNOS and nonspecific IgG expression and localization in human artery tissues from control and atherosclerotic vessels. (J) Semi-quantitative analysis of immunostaining using Image-Pro plus software n = 6 per group. Data are presented as the mean ± SE. *^, #,^ P < 0.05, **P < 0.01, ***P < 0.001 vs control group. L, lumen; M, media. Scale bar, 100 μm. (For interpretation of the references to colour in this figure legend, the reader is referred to the Web version of this article.)

In a final attempt to determine the clinical importance of our finding, two series of experiments utilizing human tissue were performed. First, we evaluated the expression levels of CREG and DNMT3B in atherosclerotic arteries from patients with arteriosclerosis obliterans who had undergone amputation and control arteries from amputees without arteriosclerosis. Representative immunofluorescence staining showed that CREG expression were lower, while DNMT3B levels were higher in the intimal and medial layers of arteriosclerotic arteries compared with the levels in control arteries (Fig. 6A and Supplement Fig. 7). A quantitative analysis demonstrated that CREG expression levels were negatively correlated with DNMT3B levels in human artery tissue, particularly in the endothelium (Fig. 6B and C and Supplement Fig. 7). To confirm the extent of methylation in *CREG* (+200/+255 bp) from arteries, we also conducted pyrosequencing using DNA extracted from atherosclerotic arteries and control arteries. Similarly, most of 12 CG sites (D1 to D5, D9, D11 and D12) in this *CREG* promoter fragment exhibited hypermethylation in atherosclerotic arteries relative to control arteries (Fig. 6D and Supplement Fig. 8).

Second, endothelium-dependent vasorelaxatory dysfunction in vascular tone is a primary process that contributes to the development of atherosclerosis [24], and is regulated by NO production and bioactivity. Thus, we assessed whether there was an association between CREG expression and vasorelaxatory function in normal and atherosclerotic arteries. Compared with normal aortic rings, human atherosclerotic aortic rings exhibited an impairment in Ach-induced relaxation (Fig. 6E). Meanwhile, NO concentrations were significantly lower in atherosclerotic arteries than those in healthy arteries (Fig. 6F). Furthermore, immunofluorescent staining and quantitative analysis showed that ROS production was dramatically elevated in atherosclerotic vessels relative to controls (Fig. 6G and H). In contrast, p-eNOS levels were significantly reduced in atherosclerotic endothelium compared with normal arteries (Fig. 6I and J).

## 4 Discussion

In the present study, we report, for the first time, that DNMT3B directly induces hypermethylation of the CREG promoter at CG (+201/+202 bp) to block the binding of GR-α to its recognition site and inhibit the expression of CREG *in vitro*. Our results also showed a negative correlation between CREG expression levels and DNMT3B levels in atherosclerotic arteries or in healthy individuals. Finally, we showed that ox-LDL reduces the expression of CREG in HUVECs by upregulating DNMT3B expression levels, and so, downregulating DNMT3B is a potential therapeutic target for the treatment of atherosclerotic endothelial dysfunction.

Previous studies have reported that epigenetic mechanisms may contribute to atherosclerosis pathogenesis and could account for some of the missing heritability in atherosclerotic cardiovascular disease [25]. Epigenetic control of transcription results in heritable changes in gene expression, without any alterations in the DNA sequence [26]. DNA methylation of promoters within CpG islands correlates with a condensed chromatin structure and leads to gene silencing, either by directly inhibiting the interaction of TFs or by attracting of methylated DNA-binding proteins, which recruit repressive complexes. The transcriptional activity of genes with CpG islands in their promoters is inversely correlated with their DNA methylation levels [27].

DNMTs are crucial in maintaining endothelial cell integrity, promoting smooth muscle cell proliferation, and inducing arteriosclerosis in animal models. These enzymes, which influence DNA methylation in vascular cells, could be used to develop new diagnostic tests and treatments for atherosclerosis-related diseases. Previous studies have reported that ox-LDL regulates gene expression by influencing DNA methylation patterns [28,29]. Moreover, the results of a previous *in vitro* cell culture study suggested that CREG expression is epigenetically regulated by DNA methylation [10]. However, neither the pathological relevance of potential DNA methylation-mediated CREG gene suppression nor the specific methylation sites have been previously investigated.

We first showed that the overexpression of DNMT3B leads to the hypermethylation of multiple CpG sites in the *CREG* promoter and that ox-LDL upregulates *DNMT3B* transcription and downregulates *CREG* transcription, consistent with previous observations that a modest increase in ox-LDL levels upregulates *DNMT1* and *DNMT3B* transcription [30].

The discovery of the effects of the *CREG* promoter DNA methylation status on GR-α binding is a novel finding and indicates the complexity of genetic and epigenetic regulatory mechanisms. GR is a member of the nuclear receptor family that controls many distinct gene networks, governing various aspects of development, metabolism, inflammation, stress responses, and other key biological processes in the cardiovascular system. GR has several receptor isoforms, for example, GRα and GRβ, which differ only in their C-termini as a result of differences in the splicing of exon 9 [31]. Some data support an important role of endogenous corticosterone via endothelial GR in reducing vascular inflammation [32], but it is less clear how GR-mediated pathways contribute to atherosclerosis.

In our study, we found that the expression of CREG was dramatically increased in HUVECs infected with viruses that overexpressed GR-α. Based on our data, we propose that *CREG* promoter activity can be regulated by both GR-α-dependent and GR-α-independent mechanisms. The interaction between epigenetic modifications and TFs is a general and important mechanism of gene expression regulation. More importantly, we demonstrated that 12 CG sites in the CREG promoter fragment (+200 to +255bp) exhibited hypermethylation, increased DNMT3B expression, and reduced CREG expression in atherosclerotic arteries from human patients compared with healthy controls. Atheroprotective ER methylation has been reported to be increased in atherosclerosis plaques relative to healthy proximal aorta [33],supporting our findings.

There are several limitations to the current study. Clearly, due to a limited source of cadavers, the quantity of the available human coronary samples is relatively low; Furthermore, DNMT3B is ubiquitously expressed, and is not limited to endothelial cells. It was recently shown that the anti-atherosclerotic effect of DNMT inhibition by 5-Aza-dC also has a mechanistic basis in the immune milieu [34]. Because atherosclerosis is a systemic disease caused by the dysfunction of multi-cell types, including immune cells, endothelial cells, and smooth muscle cells, a systemic approach will be necessary to understand the role of global methylation changes and crosstalk between cell types in modulating disease development. Moreover, the therapeutic potential of epigenetic interventions is going to be perceived. It is foreseeable that epigenetic targets such as DNMTs, specific gene promoter DNA methylation, histone modifications, and miRNAs, could serve as biomarkers for the diagnosis of cardiovascular disease and as targets for therapeutic intervention. Finally, this study lacks evidence about the in vivo cause-effect relationship between DNA hypermethylation and endothelial dysfunction in the setting of atherosclerosis. In our previous study, we detected the mouse CpG island is located in the mCREG promoter -74/+440. Although the key regulatory region located at +182/+385 was determined by using the designed reporter vectors, we did not observed analogously methylated sites binding to GR-a, which resulted in our determining that the mouse model is not suitable for to verifying the mechanism of the regulation of hCREG methylation in vivo (data are now shown). This finding demonstrates that the tissue and species specificity of DNA methylation patterns are important to consider when investigating the epigenetic mechanisms of disease [35].

**Fig. 7:**
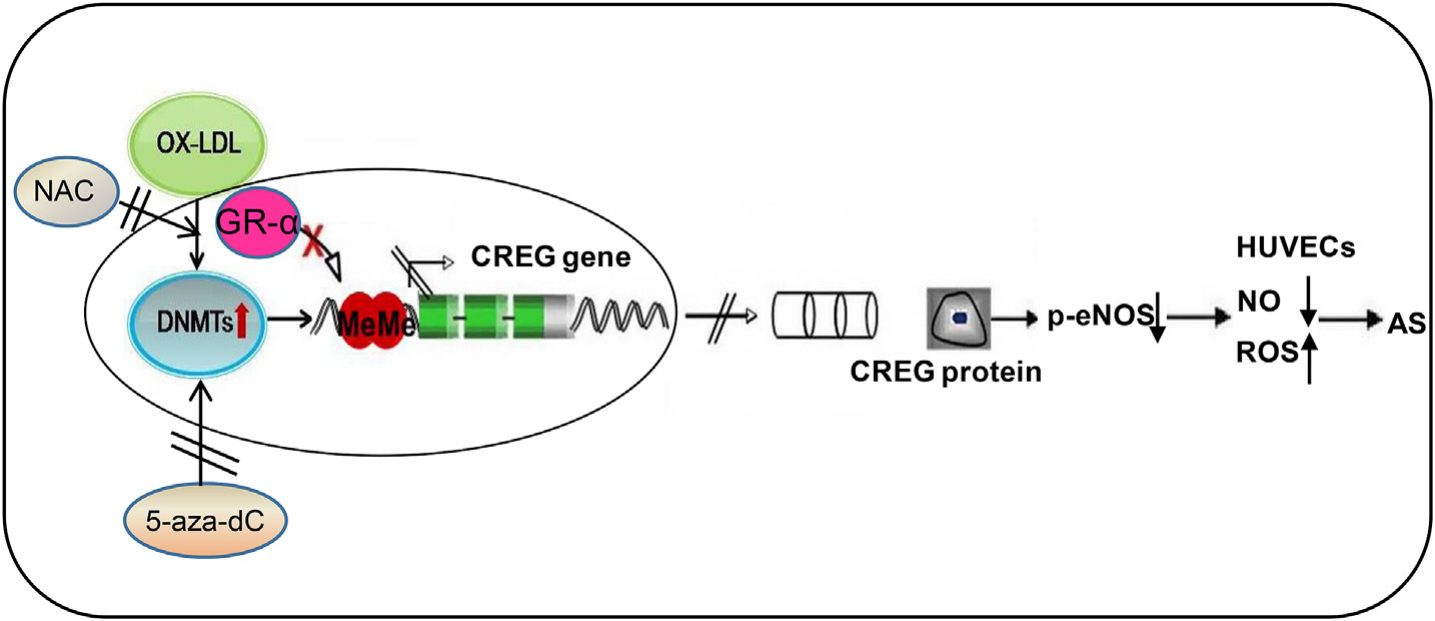
**Schematic illustration of the mechanism.** DNA hypermethylation of CG (+201/+202 bp) in the CREG promoter, induced by ox-LDL, inhibits the binding of GR-α, resulting in reduction of CREG transcription, thus contributing to endothelial diastolic function injury via the p-eNOS pathway. NAC or 5-aza-dC could reverse this effect on CREG transcription thereby attenuating the damage to endothelial diastolic function.

In summary, our findings suggest that demethylating drugs, such as 5-aza-dC or the anti-oxidative molecule NAC, can directly rescue the ox-LDL-induced reduction in CREG expression through demethylation within the CREG regulatory region, which then activates NO synthesis and improves diastolic endothelial function through the molecular activation of p-eNOS (Fig. 7).

## 5 Conclusion

In conclusion, our data suggest that DNA hypermethylation of the CG dinucleotide at +201/+202 bp of the CREG promoter, induced by ox-LDL, may inhibit binding of GR-α, resulting in the reduction of CREG transcription, and contributing to endothelial diastolic function injury via the p-eNOS pathway. Demethylating drugs such as 5-aza-dC or anti-oxidative molecule NAC could reverse the inhibition of CREG transcription thereby attenuating endothelial diastolic function injury.

## Disclosures

None.

## Declaration of competing interest

This manuscript is not any conflicts of interest.
