# Supplementary material for: DNA hypermethylation: A novel mechanism of CREG gene suppression and atherosclerogenic endothelial dysfunction
Source: Redox Biol. 2020 Jan 31;32:101444. doi: 10.1016/j.redox.2020.101444 (PMC7264464; doi:10.1016/j.redox.2020.101444)
Supplement: Multimedia component 2 [file mmc2.pdf]

Supplemental Table 1.

Nucleotide sequences of the forward and reverse primers for RT-PCR.

Gene name	Primers
CREG	Forward: GGCGTGCCCTATTTCTACCTG
	Reverse: TTTCTTGCAGAAGTTGGTCTGT
DNMT1	Forward: AAGAACGGCATCCTGTACCGAGTT
	Reverse: TGCTGCCTTTGATGTAGTCGGAGT
DNMT3A	Forward:TTTGAGTTCTACCGCCTCCTGCAT
	Reverse:GTGCAGCTGACACTTCTTTGGCAT
DNMT3B	Forward:AGTGTGTGAGGAGTCCATTGCTGT
	Reverse:GCTTCCGCCAATCACCAAGTCAAA
GR-α	Forward:AATCAGACTCCAAGCAGCGAAGAC
	Reverse:GGCAGCAGACACAGCAGTGG
GAPDH	Forward:CCAGGCGCCCAATACG
	Reverse:CCACATCGCTCAGACACCAT

Supplemental Table 2.

A

CREG sequence(+74/+255bp) modified by bisulfite (C was convertd to T except C in CG sites).

sequence modified by bisulfite (C was convertd to T except C in CG sites)

```
1    GTAAAGAGGTTGGTTTTTTTTTAGTAGTACGATTTGGGTAAGTTGTTTGATTTTTTTAAG
61   TTTTTTTTTTTTCGTTTGTTAAATAAAATAAAATAAATATTTCGTTTAGGATAAATTTAT
121  AATTTTTAAAGAGGGTAGGTAGAGCGATTGGAATGTAGATGAATTAATTTTTTTTTTTGT
181  AGGTTGCGGAGTTGTAGAGGGATTTCGTTTTTTGTATTGTGTTTAGTTTTGATTGGCGTT
241  TCGCGGAGGGGGCGGGGTTTGGGCGCGTCGAGTTTCGGTTGGGTTTTTGTAGGTTTTGGG
301  GTTCGGGATTTTTTTTGGAGATATCGTTATGGTCGGGTTATTTCGCGGGTTCGCGCGCGT
361  ATTGTTCGTCGTTTTGTTGGCGTCGACGTTGTTGGCGTTGTTCGTGTCGTTCGCGCGGGG
421  TCGCGGCGGTCGGGATTACGGGGATTGGGACGAGGTTTTTCGGTTGTCGTCGTTATTATT
481  TCGCGAGGACGCGGCGCGCGTGGTTCGTTTCGTGACGTACGTTTTCGATTGGGGCGTTTT
541  GGTTATTATTTTTACGTTGGAGGCGGTGCGCGGTCGGTTTTTCGTCGACGTTTTTTCGTT
601  TAGCGACGGGTTTTCGGGCGCGGGTAGCGGCGTGTTTTATTTTTATTTGAGTTCGTTGTA
```

B

Primers for PCR and sequence

PCR primer-F	159-1F	GGTTAGGGATTTTTTTTGGAGA	299 to 458= 159bp
PCR primer-R	159-1R	AAACCTCTTCCCAATCCCCTTAATC 5´	
Sequencing primer	159-1FS	GTTAGGGATTTTTTTTGGAGATA	
	Target sequence	TYGTTATGGTYGGGTTATTTYGYGGGTTYGYGYGYGTATTGTTYGTYGT	
PCR primer-F	161-2F	GGTTAGGGATTTTTTTTGGAGA 5’	299-460= 161bp
PCR primer-R	161-2R	AAAAACCTCTTCCCAATCCCCTTAAT	
Sequencing primer	161-2RS	TCCCAATCCCCTTAATCC（backward sequencing）	
	Target sequence	CRACCRCCRCRACCCCRCRCRAACRACACRAACAACRCCAACAACRTCRACRCC	
PCR primer-F	235-3F	TGGAGATATYGTTATGGTYGGGTTA	316-551= 235bp
PCR primer-R	235-3R	AAATAATAACCAAAACRCCCCAATC 5’	
Sequencing primer	235-3FS	GGGATTAAGGGGATTGGGA	
	Target sequence	YGAGGTTTTTYGGTTGTYGTYGTTATTATTTYGYGAGGAYGYGGYGYGYGTGGTTYGTT	

Supplemental Figure 1.

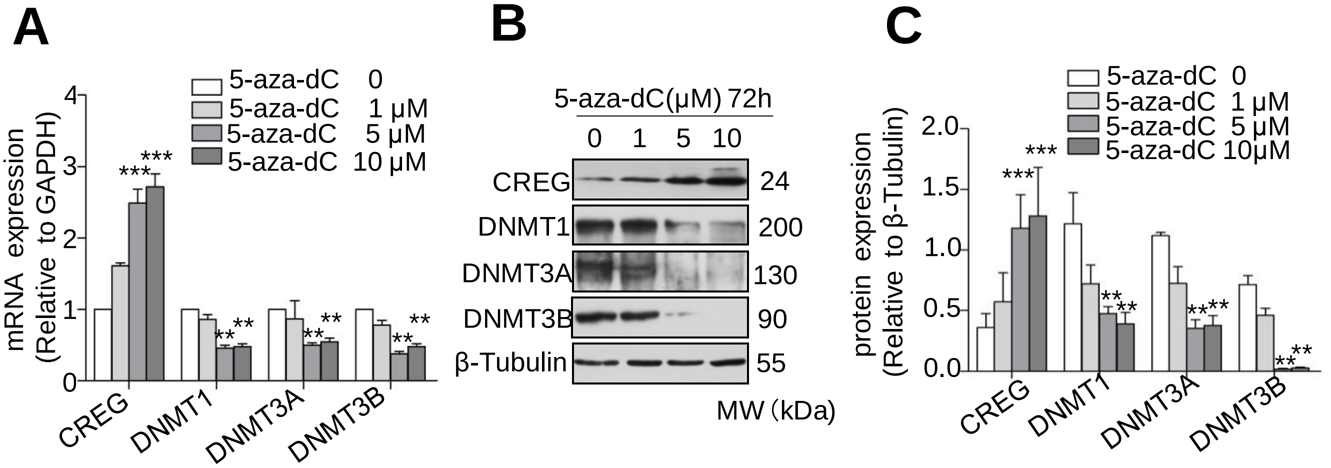

Supplemental Figure 1.CREG expression is upregulated by 5-aza-dC in HUVECs. (A) Quantitative real-time PCR, **(B)** western blotting, and **(C)** quantification of CREG, DNMT1, DNMT3A, and DNMT3B in primary HUVECs treated with various concentrations of 5-aza-dC. Specific proteins were quantified in western blots using Image-Pro plus software. Data are presented as the mean ± SE, n=6 per group, **P < 0.01 and ***P < 0.001) vs. controls (5-aza-dC 0 groups); two-sided Student's t test.

Supplemental Figure 2.

A

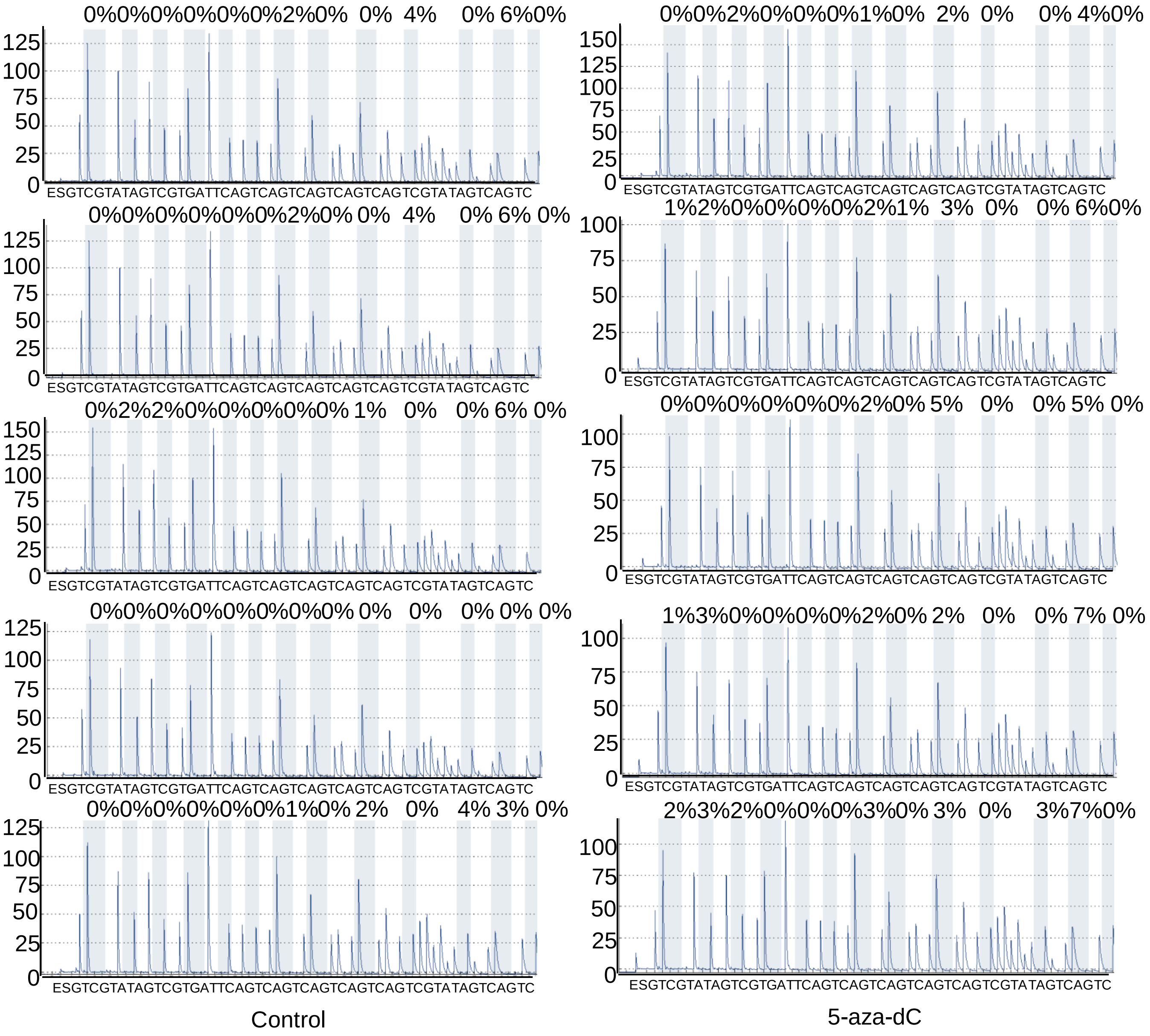

Supplemental Figure 2.(A) HUVECs were treated with 5 µM 5-aza-dC for 72h. Genomic DNAs were extracted and measured methylation levels at the +74/+128bp via pyrosequencing. There is no significant difference in the two groups (n=5).

Supplemental Figure 2.

B

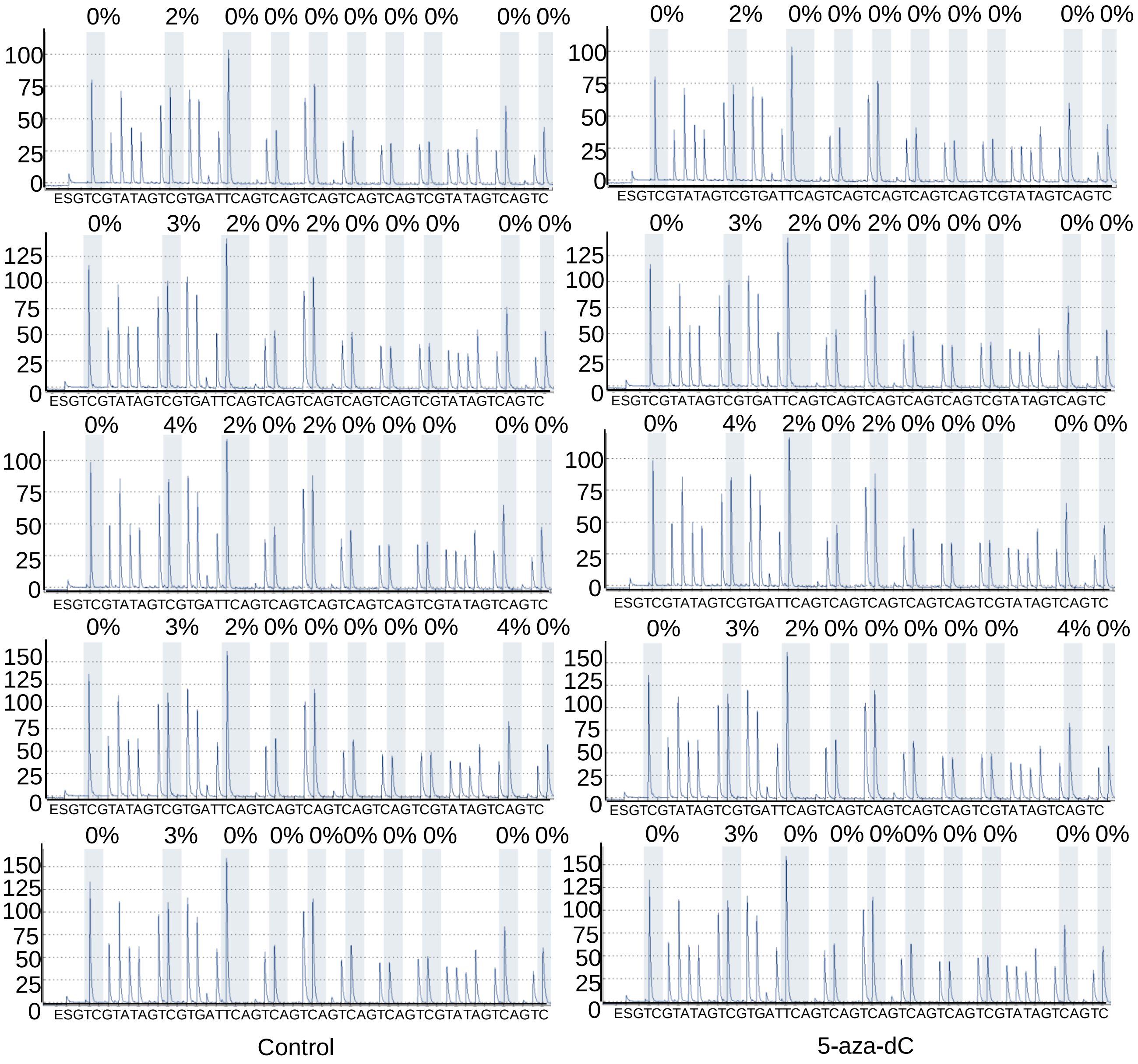

Supplemental Figure 2.(**B**) HUVECs were treated with 5 µM 5-aza-dC for 72h. Genomic DNAs were extracted and measured methylation levels at the +129/+199bp via pyrosequencing. There is no significant difference in the two groups (n=5).

Supplemental Figure 2.

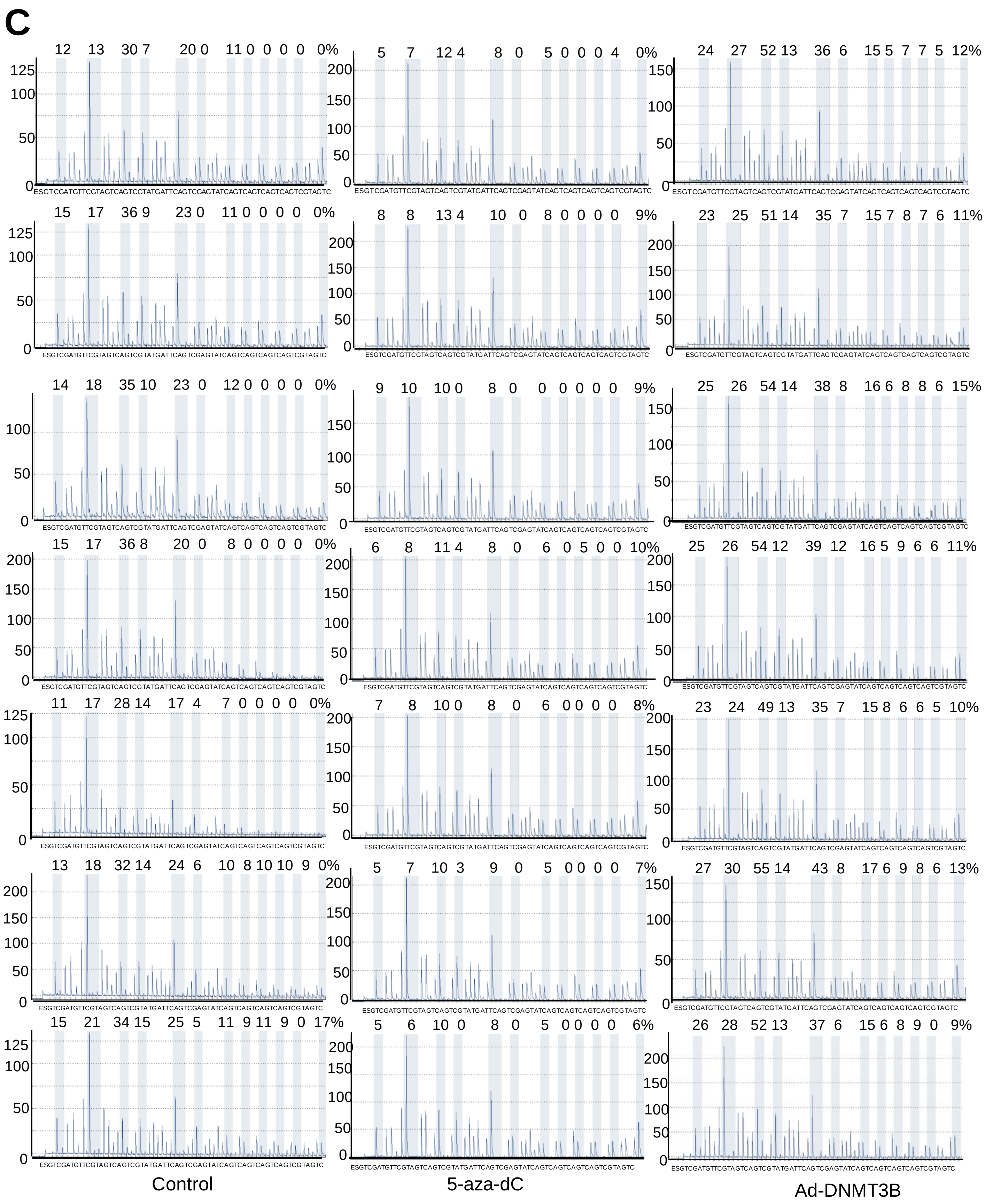

Supplemental Figure 2.(**C**) HUVECs were treated with 5 µM 5-aza-dC for 72h or 50MOI Ad-DNMT3B for 48 h. Genomic DNAs were extracted and measured methylation levels at the +200/+255bp via pyrosequencing (n=7).

Supplemental Figure 3.

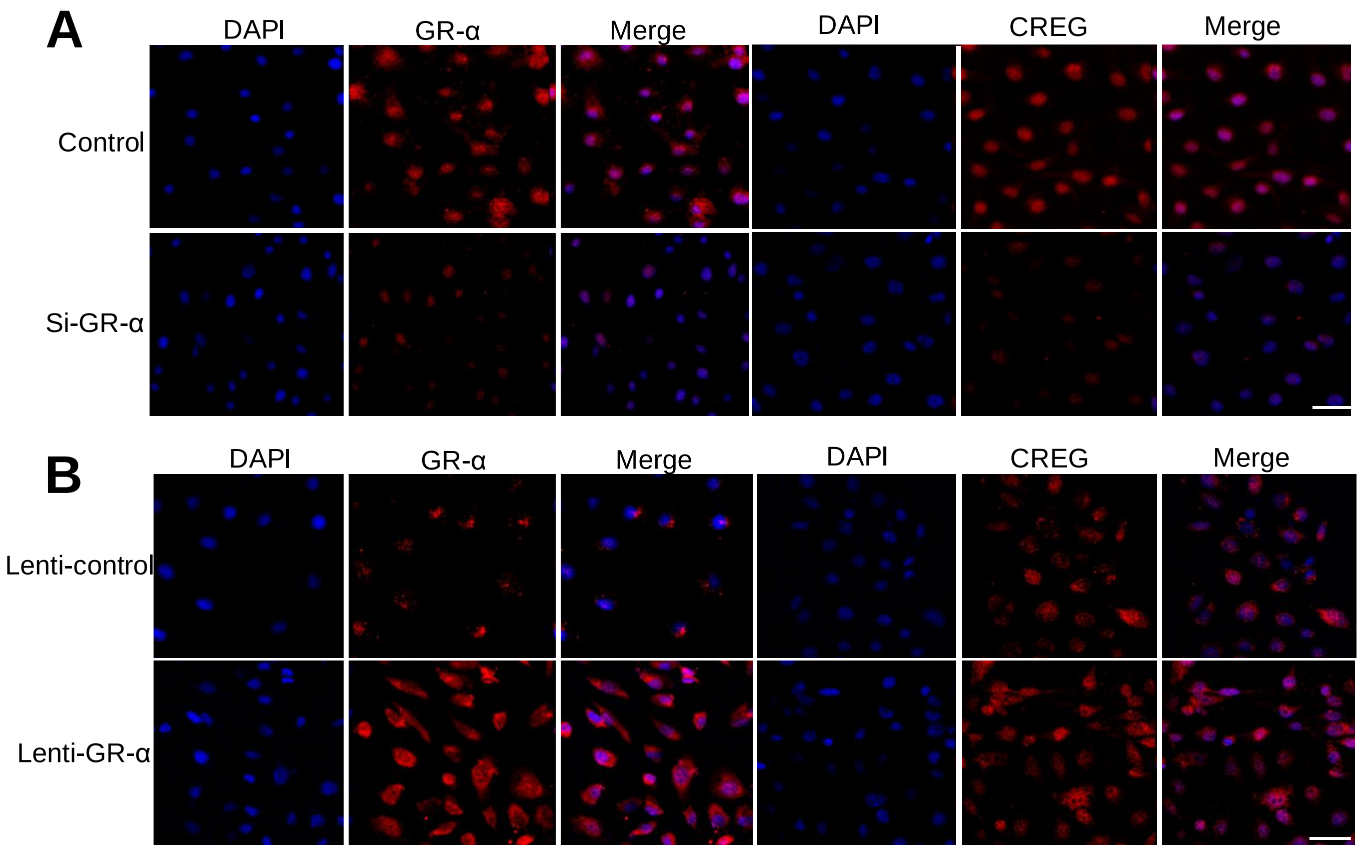

Supplemental Figure 3. The effect of SiRNA or lentivirus transduced GR-α influence CREG levels in HUVECs. (**A**) Immunofluorescence staining show that expression of CREG were decreased with Si-GR-α **(B)** Expression of CREG were increased with lenti-GR-α. Scale bar, 100μm.

Supplemental Figure 4.

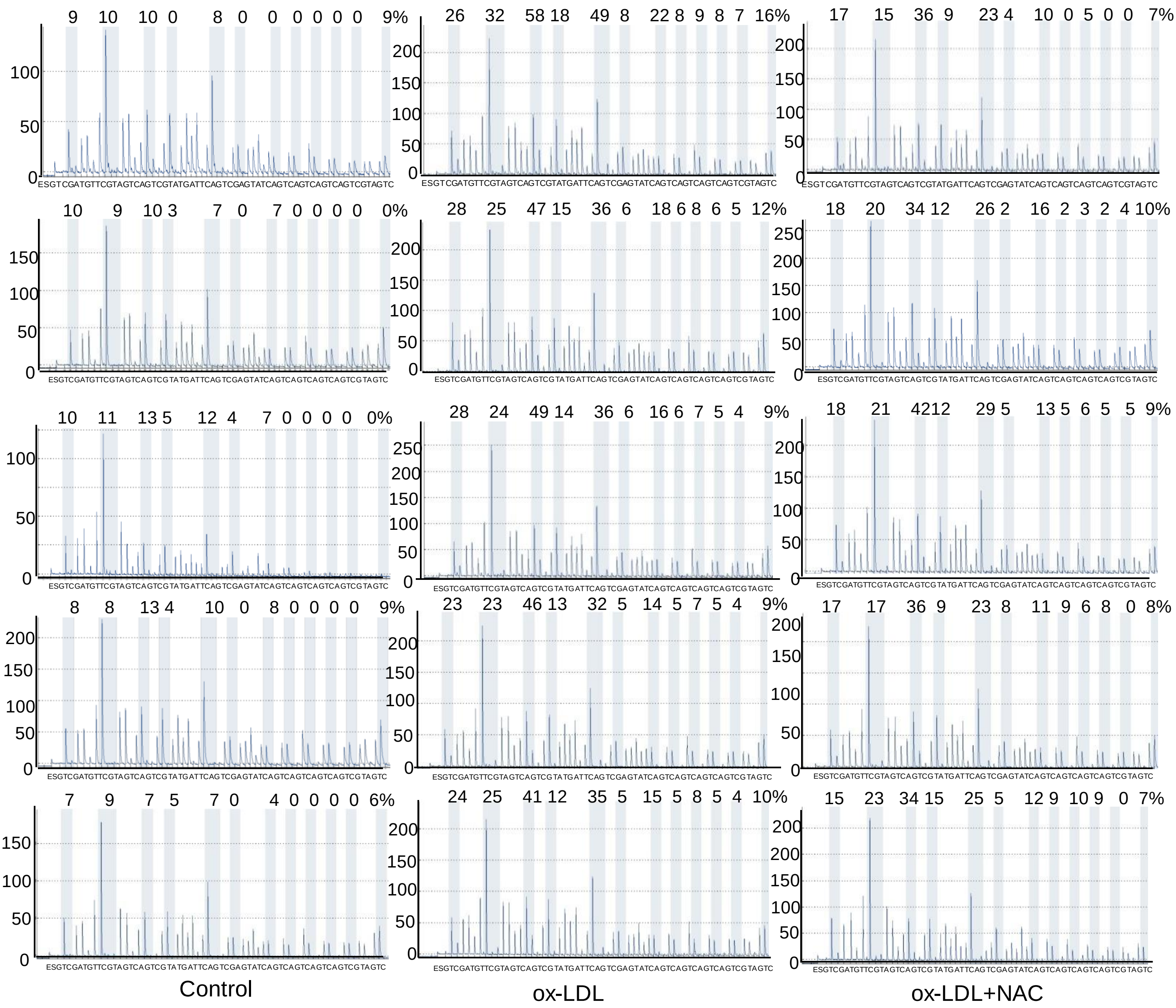

Supplemental Figure 4. HUVECs were treated with 40 μg/ml ox-LDL for 24h and in the presence of 1mM NAC for 24 h (n = 5). Genomic DNAs were extracted and measured methylation levels at the +200/+255bp via pyrosequencing (n=5).

Supplemental Figure 5.

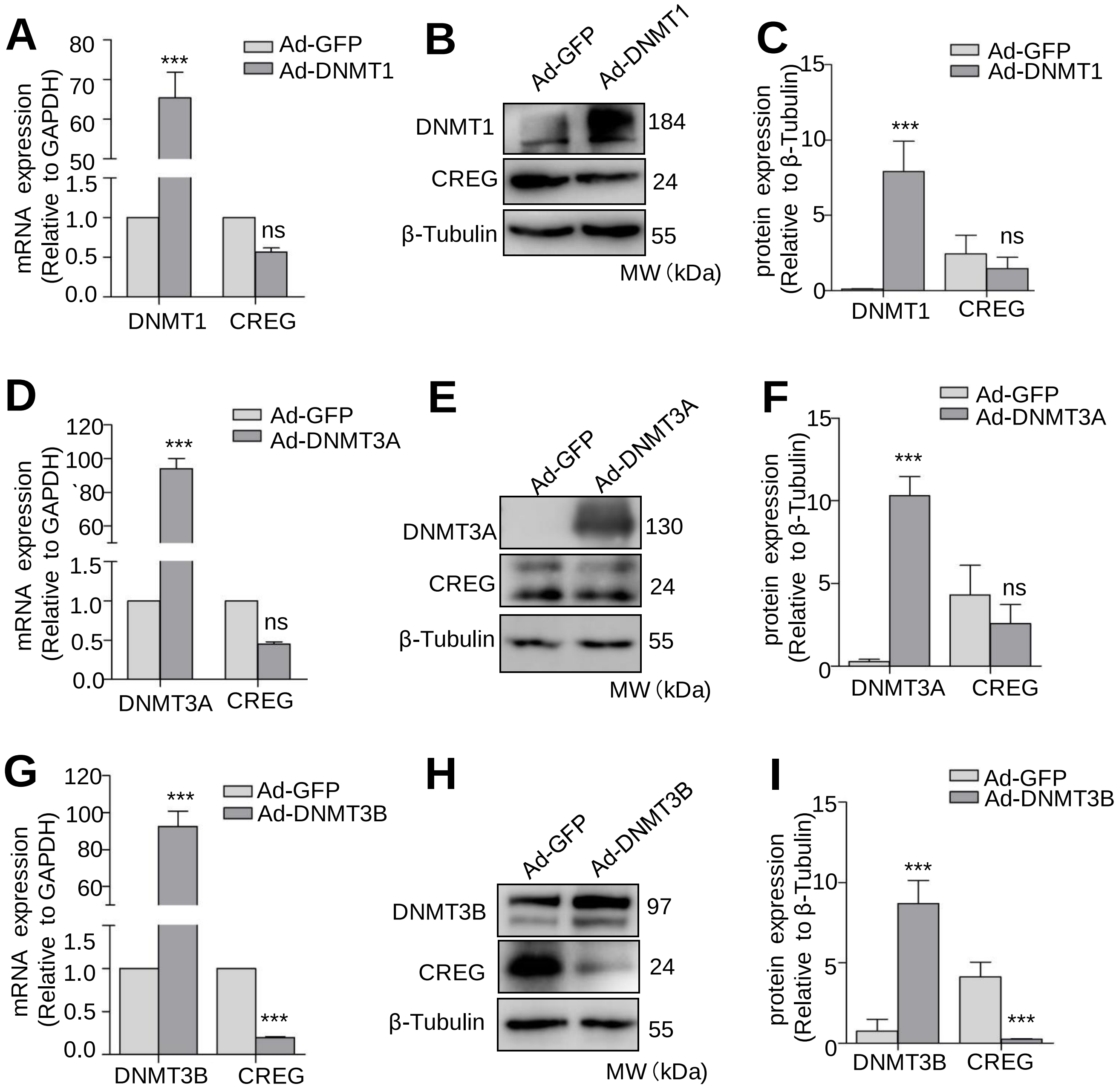

Supplemental Figure 5. DNMT3B directly regulates the expression of CREG in HCAECs. (A to I) Quantitative real-time PCR (A, D, G), representative western blotting (B, E,H), and quantification analysis (C, F, I) of CREG, DNMT1, DNMT3A and DNMT3B in HCAECs after infection with Ad-DNMT1, Ad-DNMT3A, and Ad-DNMT3B for 48 h respectively. Specific proteins were quantified in western blots using Image-Pro plus software. Data are presented as the mean ± SE. ns, no significant difference, ***P < 0.001 vs. Ad-GFP; n=3 per group, two-sided Student's t test.

Supplemental Figure 6.

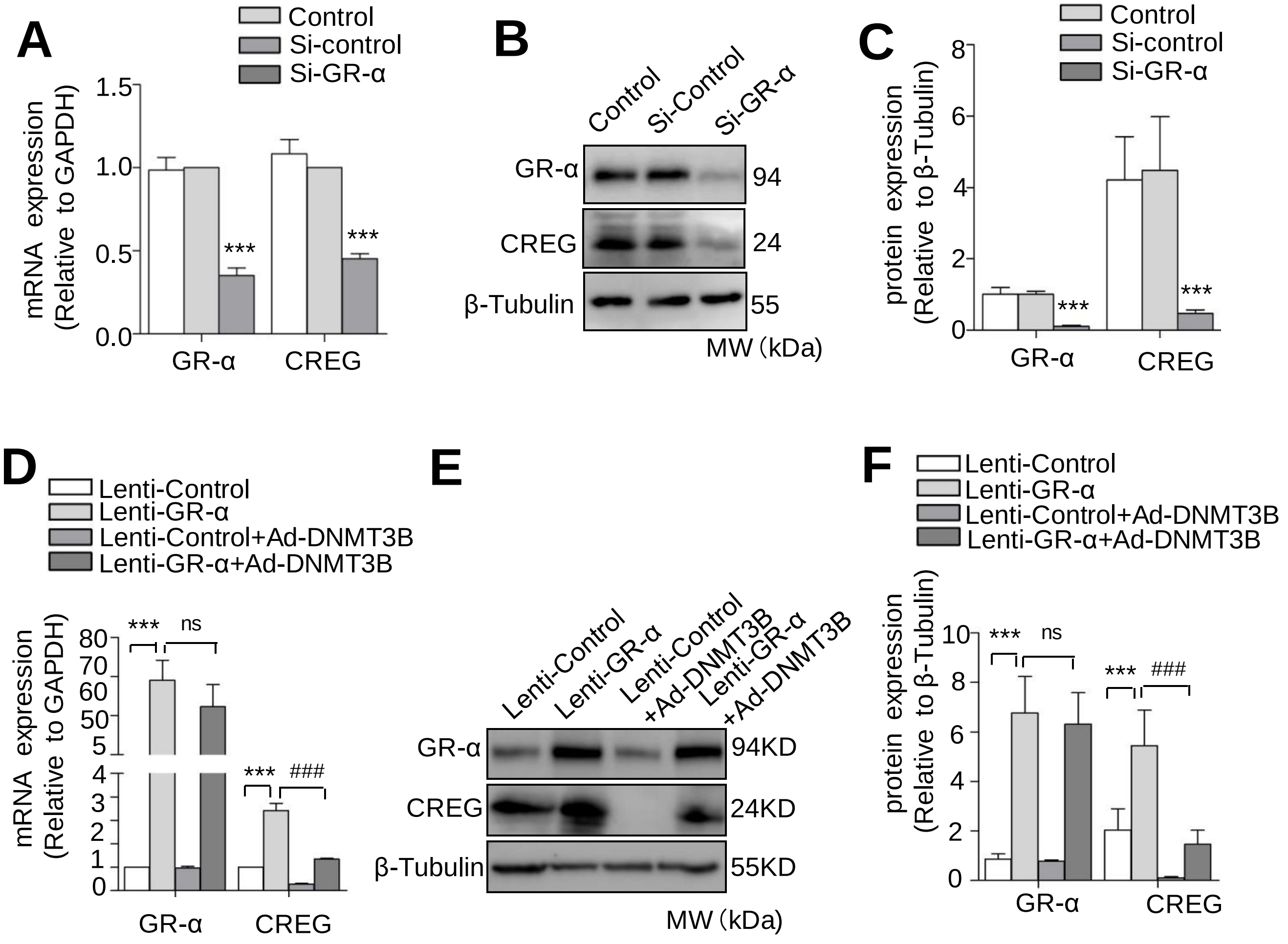

Supplemental Figure 6. (A) Quantitative real-time PCR, **(B)** representative western blotting, and **(C)** quantification in HCAECs with GR-α knocked down using siRNA (Si-GR-α). **(D)** Quantitative real-time PCR, **(E)** western blotting, and **(F)** quantification in primary HCAECs overexpressing GR-α (Lenti-GR-α) with or without Ad-DNMT3B infection. Quantification of western blots was conducted using Image-Pro plus software. Data are presented as the mean ± SE, n=3 per group, ***, ### *P* < 0.001 vs. Lenti-control, or Si-control groups.

Supplemental Figure 7.

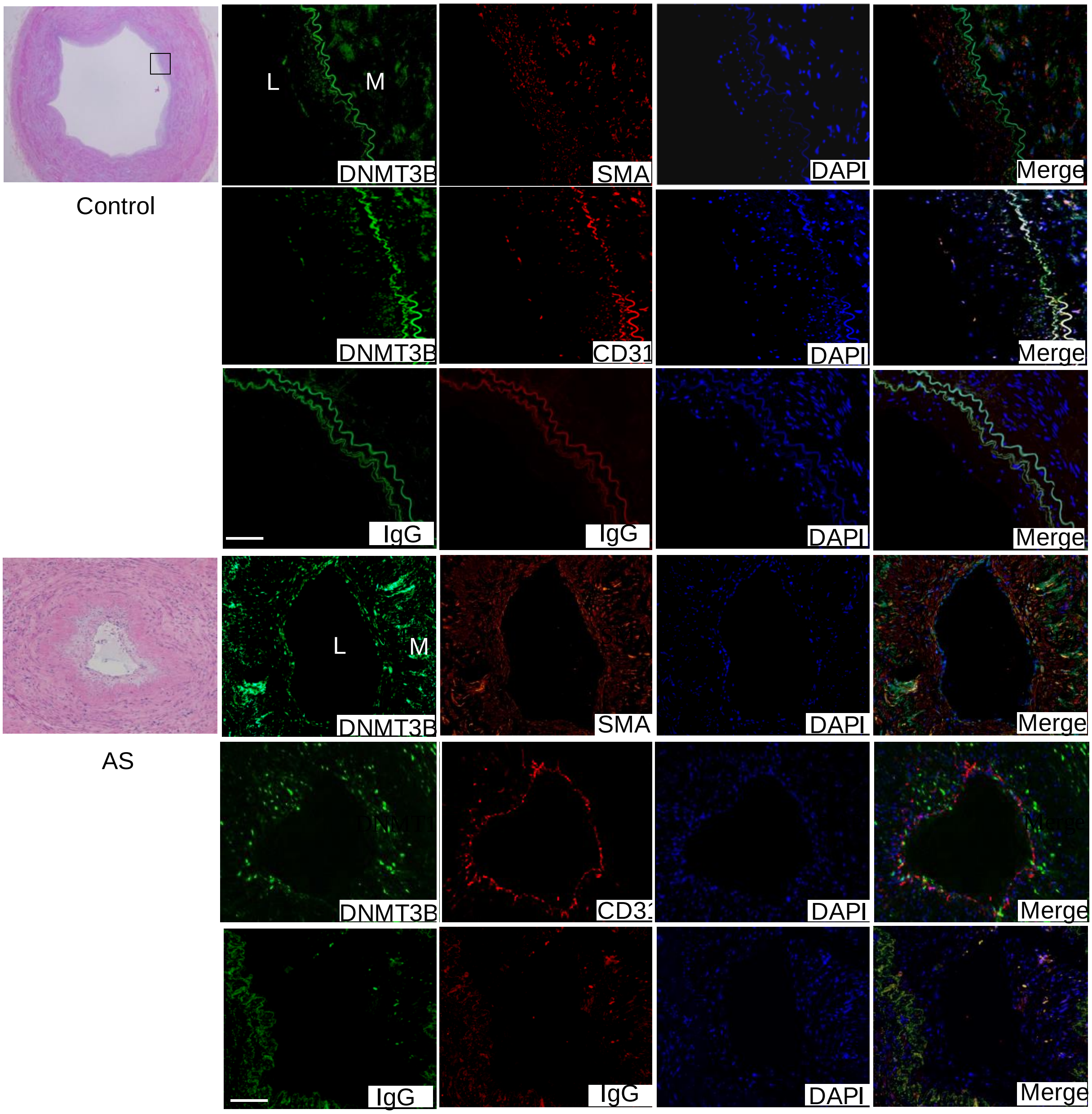

Supplemental Figure 7. Expression of DNMT3B were increased in atherosclerotic arteries relative to the results in the normal arteries whether in endothelial cells or smooth muscle cells. L, lumen; M, media Scale bar, 100μm.

Supplemental Figure 8.

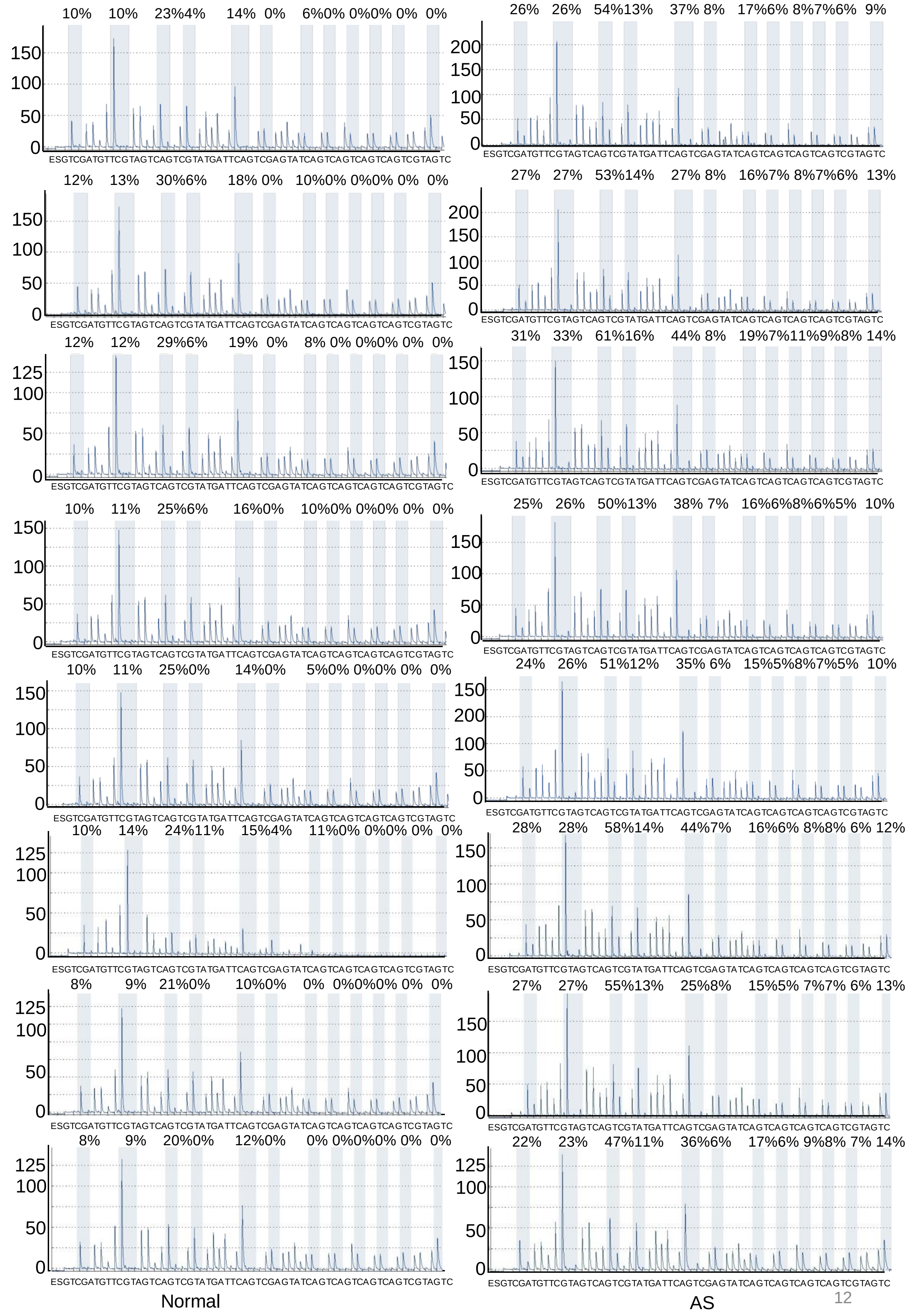

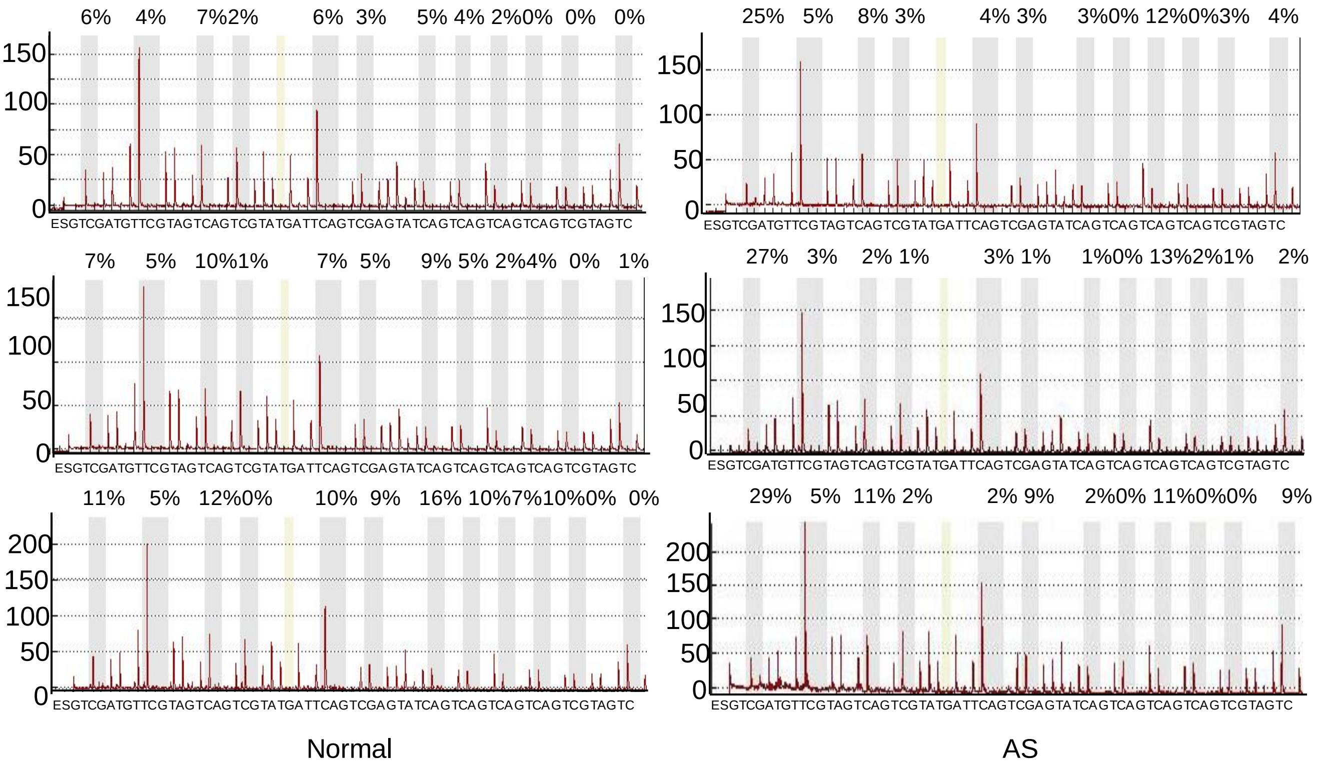

Supplemental Figure 8. Genomic DNAs were extracted from atherosclerotic arteries and normal arteries(including 8 low extremity and 3 coronary atherosclerosis per group) then measured methylation levels at the +200/+255bp via pyrosequencing（n=11).
